# Ecological plasticity of *Halanaerobium* microorganisms across terrestrial saline to hypersaline subsurface environments

**DOI:** 10.1128/spectrum.01381-26

**Published:** 2026-06-15

**Authors:** Yu He, Xinhang Wang, Shuyi Li, Chenxi Zhang, Mingxia Xu, Yushan Zhou, Robert A. Sanford, Renxing Liang, Yanqun Zhu, Dongbo Yang, Lihua Dan, Xingdong Mao, Lei Zhang, Weimin Sun, Yongguang Jiang, Yidan Hu, Zhou Jiang, Yongzhe Li, Wentao Song, Na Hu, Lu Zhao, Yiran Dong, Liang Shi

**Affiliations:** 1 School of Environmental Studies, China University of Geosciences 12564, Wuhan, China; 2 School of Environment and Resources, Taiyuan University of Science and Technology 117763, Taiyuan, China; 3 Department of Earth Science & Environmental Change, University of Illinois Urbana-Champaign 14589, Urbana, Illinois, USA; 4 State Key Laboratory of Geomicrobiology and Environmental Changes, China University of Geosciences 12564, Wuhan, China; 5 Sinopec Zhongyuan Oilfield 244023 , Puyang, Henan, China; 6 Guangdong Institute of Eco-environmental and Soil Science 370585 , Guangzhou, Guangdong, China; 7 Central and South China Municipal Engineering Design and Research Institute Co, Ltd., Wuhan, China; 8 State Environmental Protection Key Laboratory of Source Apportionment and Control of Aquatic Pollution, Ministry of Ecology and Environment 251406 , Wuhan, China; 9 Hubei Key Laboratory of Yangtze Catchment Environmental Aquatic Science, Wuhan, China; 10 Key Laboratory of Groundwater Quality and Health (China University of Geosciences), Ministry of Education 12564, Wuhan, China; Connecticut Agricultural Experiment Station, New Haven, Connecticut, USA

**Keywords:** deep biosphere, *Halanaerobium*, sulfide generation, comparative genomics, ecological plasticity

## Abstract

Members of the genus *Halanaerobium* are widely distributed in hypersaline environments, including oil and gas reservoirs, and saline lake sediment. However, a comprehensive understanding of their physiological traits, metabolic capacities, adaptive strategies, and biogeography remains limited. In this study, a strictly anaerobic and halophilic strain, *H. saccharolyticum*_B KY39 was isolated from produced water in the Zhongyuan Oilfield, China. Strain KY39 grew at 20–45°C, 2–30% salinity, pH 5.3–9.0, and up to 50 MPa hydrostatic pressure. It could ferment various carbohydrates (e.g., glucose, xylose, sucrose, and maltose) or use mannitol and pyruvate as electron donors under Fe(III)-reducing conditions. Comparative genomic analyses of 31 high-quality *Halanaerobium* strains revealed an open pangenome. Genes involved in osmotic and pressure stress responses, including those related to osmoprotectant biosynthesis and ion transport, were highly conserved. The thiosulfate sulfurtransferase (TST) gene, responsible for converting thiosulfate to sulfite, was universally present. Notably, compared to the strains from saline lakes, those from oil and gas reservoirs possessed larger genomes and harbored a broader repertoire of genes related to peptidoglycan biosynthesis, nitrogen fixation, sulfur metabolism, biofilm formation, and carbohydrate uptake, suggesting enhanced metabolic flexibility and environmental adaptation. Moreover, a survey of the available metagenomes revealed that *Halanaerobium* species were globally distributed across diverse environments exhibiting a broader salinity range. In addition to oil and gas reservoirs and saline lakes, they also widely reside in soils, fermented foods, and marine ecosystems. Collectively, these findings advance the systematic understanding of ecological plasticity and metabolic versatility of *Halanaerobium*, shedding light on their ecological roles and potential industrial impacts.

**IMPORTANCE:**

Members of the genus *Halanaerobium* are prominent inhabitants of surface and deep subsurface hypersaline environments, yet their ecological roles and adaptive strategies remain poorly understood. Here, through the isolation of a novel strain from the production fluid of an oil field combined with comparative genomic analyses across the genus, we revealed the metabolic versatility, stress tolerance, and global distribution of *Halanaerobium*. Our findings underscore the ecological plasticity, functional diversity, and niche differentiation within this genus, providing fundamental insights into its potential industrial and environmental applications.

## INTRODUCTION

The terrestrial deep subsurface, extending several kilometers beneath Earth’s surface, represents a vast, light-independent biosphere and one of the largest habitats on the planet (1). Its total biomass is estimated to be comparable to that of global surface soils, accounting for approximately 12–20% of Earth’s total biomass (2–4). Due to its immense scale and unique geochemical conditions (e.g., elevated temperature, pressure, and salinity), this ecosystem plays an essential central role in global biogeochemical cycles, particularly in regulating carbon, sulfur, and other elemental fluxes (5). In addition, terrestrial deep subsurface environments serve as geochemical analogs for saline-alkaline subsurface habitats on Mars and other planetary bodies, offering key insights into the potential for extraterrestrial life (6, 7). Elucidating the structure and function of this biosphere is critical for understanding the origin, adaptation, and evolution of life, as well as for its significant impacts on industrial processes, including oil and gas recovery, carbon sequestration, nuclear waste disposal, and hydrogen storage, where redox-driven immobilization of toxic metals is of particular importance (1, 8–11). Thus, the study of deep subsurface microbial communities is of both fundamental and practical significance.

The deep subsurface hosts diverse microbial communities adapted to extreme physicochemical conditions, including elevated temperature, pressure, salinity, and oxygen limitation (12, 13). These communities exhibit a broad spectrum of metabolic capabilities shaped by their geochemical conditions (5, 12). Among them, the genus *Halanaerobium* is a group of halophilic anaerobes that are widespread and particularly dominant in hydraulically fractured shale gas reservoirs (e.g., the Marcellus, Barnett, Antrim, and Haynesville formations in the United States) (12, 14–17). *Halanaerobium* exhibits remarkable ecological versatility, fermenting various carbohydrates (e.g., guar gum) and producing sulfide from thiosulfate (18). These metabolic traits, together with their capacity to form adhesive biofilms, can alter reservoir permeability, drive souring, and promote pipeline corrosion (19, 20). Moreover, their tolerance to extended exposure to biocides like glutaraldehyde underscores their resilience in engineered environments and makes them “notorious” in the oil and gas industry (19). Beyond subsurface reservoirs, *Halanaerobium* species have also been detected in and cultivated from hypersaline lakes, reflecting their broad ecological distribution (16, 21). Given their metabolic versatility, adaptability, and ecological significance, comparative genomic analyses of *Halanaerobium* offer critical insights into the genetic basis of adaptive survival and functionalities in hypersaline ecosystems.

Although the physiology and metabolic potential of *Halanaerobium* species in some saline lakes and deep subsurface environments have been explored, a systematic understanding of their adaptive evolution, metabolic diversification across phylogenetic clades, and distinct environmental origins remains limited. In this study, a *Halanaerobium* strain was isolated from the production fluids of the Zhongyuan Oilfield, China. To gain broader insights, we integrated all publicly available *Halanaerobium* genomes with the genome reconstructed in this study and performed comprehensive phylogenetic, physiological, and comparative genomic analyses. This study elucidates the ecological diversity, survival strategies, and functional potential of the *Halanaerobium* species and offers valuable insights into their environmental adaptation and potential practical applications.

## MATERIALS AND METHODS

### Enrichment and isolation

The sampling site was located at an oil production well in the Zhongyuan Oilfield, Henan, China (115.399,094°E, 35.766,964°N). Production fluid was collected in sterile bottles, stored on ice, and transported to the laboratory in September 2021 for geochemical analyses (Table S1). Measurements of geochemical parameters (e.g., pH, temperature, soluble anions, total carbon [TC], and total organic carbon [TOC]) were performed following the methods described in our previous studies (22, 23).

An anaerobic underground water medium (UGW medium) was used for the enrichment of native organisms (24). Part of the production fluid collected was promptly inoculated into the UGW medium at a 1:5 ratio on site using sterilized syringes. The UGW medium contained a mixture of small-molecule organic acids (i.e., pyruvate, formate, acetate, and lactate, 10 mM each) as carbon sources and electron donors, with 10 mM ferric citrate supplied as the electron acceptor (24). The enrichment cultures were transferred into fresh medium every 2–3 weeks, and the microbial activities were monitored by the changes in biogenic Fe(II) concentration. After three rounds of transfer, based on preliminary amplicon sequencing of the bioactive enrichment cultures, the UGW medium was optimized (optimized UGW medium, oUGW) to enhance the growth of the dominant *Halanaerobium* spp. (g L^−1^): NaCl (100.0), MgCl_2_·6H_2_O (0.33), NH_4_Cl (0.33), CaCl_2_·2H_2_O (0.33), KH_2_PO_4_ (0.33), NaHCO_3_ (1.5), peptone (5.0), cysteine (0.031), Se/Wo solution (0.5 mL L^−1^), trace metal solution (1 mL L^−1^), and vitamin solution (1 mL L^−1^) (25). The medium was degassed with high-purity N_2_ (99.9%) and sterilized by autoclaving at 121°C for 20 min.

The organism was isolated using the spread plate method by sequentially diluting the enrichment cultures into fresh oUGW supplemented with 2% (wt/vol) agar. After approximately 3 days of incubation, white, smooth, and creamy colonies were observed. The representative single colonies were picked using sterile inoculation loops and transferred into fresh liquid medium. This dilution and cultivation process was repeated three times to obtain a pure culture. The obtained isolate was designated strain KY39.

### Morphological and physiological characterization

The cell morphology of strain KY39 was examined using a Hitachi SU8010 scanning electron microscope (Hitachi, Ltd., Tokyo, Japan) following previously described pretreatment protocols (26). The aggregated cells grown under ferrihydrite-reducing conditions were sequentially stained with calcein AM and propidium iodide following the instructions of the Viability Assay Kit for Live & Dead Cells (MedChemExpress, NJ, USA). The live and dead cells were differentiated by visualization under an Olympus IX83 fluorescence microscope (Olympus Corporation, Tokyo, Japan).

The physiological traits of strain KY39 were assessed using cultures grown in oUGW medium under varying environmental conditions. Metabolic activity was assessed by measuring Fe(II) production or optical density at 600 nm (OD_600_). The temperature range and optimal temperature were determined by incubating cultures at temperatures between 4°C and 55°C. The pH range was assessed at 37°C using media adjusted to different initial pH values with the buffering compounds sodium acetate, 2-(N-morpholino)ethanesulfonic acid (MES), 4-(2-hydroxyethyl)−1-piperazineethanesulfonic acid (HEPES), tris(hydroxymethyl)aminomethane (Tris), and 2-(Cyclohexylamino)ethanesulfonic acid (CHES). Salinity tolerance of strain KY39 was tested by culturing it in media containing 0–30% (wt/wt) NaCl. Its pressure tolerance was evaluated under iron-reducing conditions inside stainless-steel high-pressure vessels (Nantong Feiyu Petroleum Technology Development Co., Ltd., Jiangsu, China) at hydrostatic pressures of 0.1, 10, 20, 30, 40, and 50 MPa. In these experiments, 10 mM small-molecule organic acids or 10 mM pyruvate were supplied as electron donors, and 10 mM ferric citrate was provided as the electron acceptor. Abiotic controls were prepared in the same manner as the bioactive samples, except without cell inoculation to assess abiotic Fe(III) reduction. Biomass was determined using the BCA Protein Assay Kit (Takara Bio Inc., Kyoto, Japan) following the manufacturer’s protocol. Biomass-normalized, initial pseudo-zero-order Fe(III) reduction rate constants (mM Fe(II) day^−1^ mg^−1^ protein) were calculated from the initial iron reduction phase, when the reaction rate was nearly constant and representative of iron-reducing activity by the organism.

The nutritional versatility of strain KY39 was further investigated by supplementing the oUGW medium (10% NaCl, 37°C) with different individual organic and inorganic substrates, including small-molecule organic acids, sugars, and alcohols. In these assays, no iron was added, and growth was monitored by OD_600_. All experiments were performed in triplicate.

To analyze cellular components, cells were harvested during the exponential growth phase. Cellular fatty acid methyl esters (FAMEs) were prepared and analyzed using the Sherlock Microbial Identification System (MIDI Inc., DE, USA) according to the standard protocol (version 6.1). Briefly, fatty acids were saponified and methylated to form FAMEs, and identified by gas chromatography (Agilent Technologies, CA, USA) using the TSBA database. Polar lipids were extracted from freeze-dried cells using a chloroform-methanol mixture (2:1, vol/vol). The lipid extracts were separated by two-dimensional thin-layer chromatography (TLC) on silica gel 60 plates (Merck KGaA, Darmstadt, Germany). The first dimension was developed in chloroform: methanol: water (65:25:4, vol/vol/vol), and the second dimension in chloroform:acetic acid:methanol:water (80:15:12:4, vol/vol/vol/vol). Lipid spots were visualized by spraying with specific staining reagents: phospholipids with molybdenum blue (blue spots), amino lipids with ninhydrin (purple spots), and total lipids with phosphomolybdic acid (green spots) (27). The compositions of whole-cell amino acids and sugars were determined using the method modified from that of Hasegawa et al. (28), in which paper chromatography was replaced with TLC performed on cellulose-coated plates (28).

### Molecular and bioinformatic analyses

Genomic DNA of strain KY39 was extracted using the MiniBEST Bacterial Genomic DNA Extraction Kit (Takara Bio Inc., Otsu, Japan). The purity and concentration of the extracted DNA were assessed using a NanoDrop One spectrophotometer and a Qubit 2.0 fluorometer (Thermo Fisher Scientific Inc., MA, USA). To evaluate the phylogeny of the isolated strain, the full-length 16S rRNA gene was amplified using the universal primer set 27F and 1492R (29). The purified PCR products were subsequently subjected to bidirectional Sanger sequencing at Sangon Biotech Co., Ltd. (Shanghai, China) using an ABI 3730xl DNA Analyzer (Thermo Fisher Scientific Inc.). The resulting nucleotide sequences were aligned against the National Center for Biotechnology Information (NCBI) database using BLASTn to determine the taxonomic identity of the strain (30).

Additionally, to obtain the genome of strain KY39, a sequencing library was constructed from the *Halanaerobium-*dominating enrichment culture using the NEBNext Ultra DNA Library Prep Kit for Illumina (New England Biolabs, MA, USA), and paired-end sequencing was performed on an Illumina HiSeq X-Ten platform at Magigene Biotechnology Co., Ltd. (Guangdong, China). The raw sequencing reads were quality-trimmed using Kneaddata v0.6.1 (https://github.com/biobakery/kneaddata), and *de novo* assembly was performed using SPAdes v3.15.1 (31). The assembled contigs were processed with MetaWRAP v1.2.1 to reconstruct metagenome-assembled genomes (MAGs) (32). The genomic features (e.g., tRNAs and rRNAs) were annotated using Prokka (33). Open reading frames (ORFs) were predicted using Prodigal v2.6.3 in metagenomic mode, and the predicted ORFs were functionally annotated against the KEGG, CAZymes, MEROPS, and HydDB databases to infer the metabolic pathways of strain KY39 (34–38).

In addition to strain KY39, a total of 38 publicly available genomes of the genus *Halanaerobium* were retrieved from NCBI and the Integrated Microbial Genomes & Microbiomes (IMG/M) databases in May 2023 (39). The genomic features, including GC content, completeness, and contamination, were assessed using CheckM2 (40). Only high-quality genomes with ≥90% completeness and ≤5% contamination (*n* = 30) were retained for downstream functional analyses (Table S2). The 16S rRNA gene sequences of these genomes, together with that of strain KY39, were used for phylogenetic analysis with IQ-TREE2, with branch supports estimated from 1,000 bootstrap replicates (41).

The pairwise average nucleotide identity (ANI) values among the genomes were calculated using the Integrated Prokaryotes Genome and Pan-genome Analysis (IPGA) pipeline (42). To further resolve their phylogenetic relationships, 120 bacterial marker genes were extracted, concatenated, and aligned for phylogenetic reconstruction using GTDB-Tk (43). A maximum likelihood tree was then constructed using IQ-TREE2 with the parameters “-T AUTO -m MFP -bb 1000,” and the resulting tree was visualized using iTOL (41, 44).

Orthologous gene clusters were identified using the Bacterial Pan Genome Analysis (BPGA) pipeline, which was also applied to construct the pangenome and model pangenome dynamics (45). Both a core genome phylogenetic tree and a pangenome tree were generated using BPGA (45). A tanglegram was constructed to compare the topologies of the two trees using SplitsTree (46, 47). Functional annotation and pathway reconstruction were performed based on the KEGG databases (48, 49). Principal coordinate analysis (PCoA) was performed on *Halanaerobium* genomes based on a Jaccard distance matrix calculated from the presence/absence of functional genes. Statistical differences between groups were assessed using permutational multivariate analysis of variance (PERMANOVA) with 999 permutations, as implemented in the “vegan” package in R (50). In addition, functional differences among *Halanaerobium* genomes from different environments were evaluated using a permutation-based differential analysis of KEGG orthologs (KOs) profiles (51). For each KO, the observed mean difference between the groups was compared with a null distribution generated from 9,999 random permutations. Two-sided *P* values were adjusted using the Benjamini-Hochberg method, and KOs with adjusted *P* values < 0.05 were considered significant. Significantly enriched KOs were mapped to KEGG pathways. Gene set enrichment analysis (GSEA) was further conducted using the fgsea algorithm, and pathway-level shifts were evaluated based on normalized enrichment scores (NES) (52).

The global distribution of *Halanaerobium* was analyzed using the online Sandpiper platform (https://sandpiper.qut.edu.au/), which implements the SingleM pipeline to query microbial communities across 707,470 publicly available shotgun metagenomes (53). SingleM identifies taxa based on 59 universal single-copy marker genes (22 Bacteria-specific, 24 Archaea-specific, and 13 shared across both domains) and assigns genus-level taxonomy using DIAMOND BLASTX hits against GTDB reference genomes. Species-level assignment requires ≥96.7% ANI over conserved 60 bp windows, and sequences not meeting this threshold are assigned at the genus level. To minimize false positives in complex matrices (e.g., soils, marine samples, and fermented foods), SingleM applies multiple filters, including low *e*-value recruitment, coverage thresholds (>0.35 × expected marker levels), removal of low-abundance artifacts, trimmed mean abundance calculations across markers (excluding the top and bottom 10%), and integration of multi-marker signals. These procedures enhance specificity across diverse environments. Detection of low-abundance taxa is limited by sequencing depth, potentially underestimating those present at <0.01–0.1% relative abundance due to insufficient marker hits. Additionally, rare contamination or sequencing artifacts may generate trace false positives, though these are largely minimized by the applied filters.

## RESULTS

### Morphological, taxonomic, and genomic characteristics of the isolate

**Fig 1:**
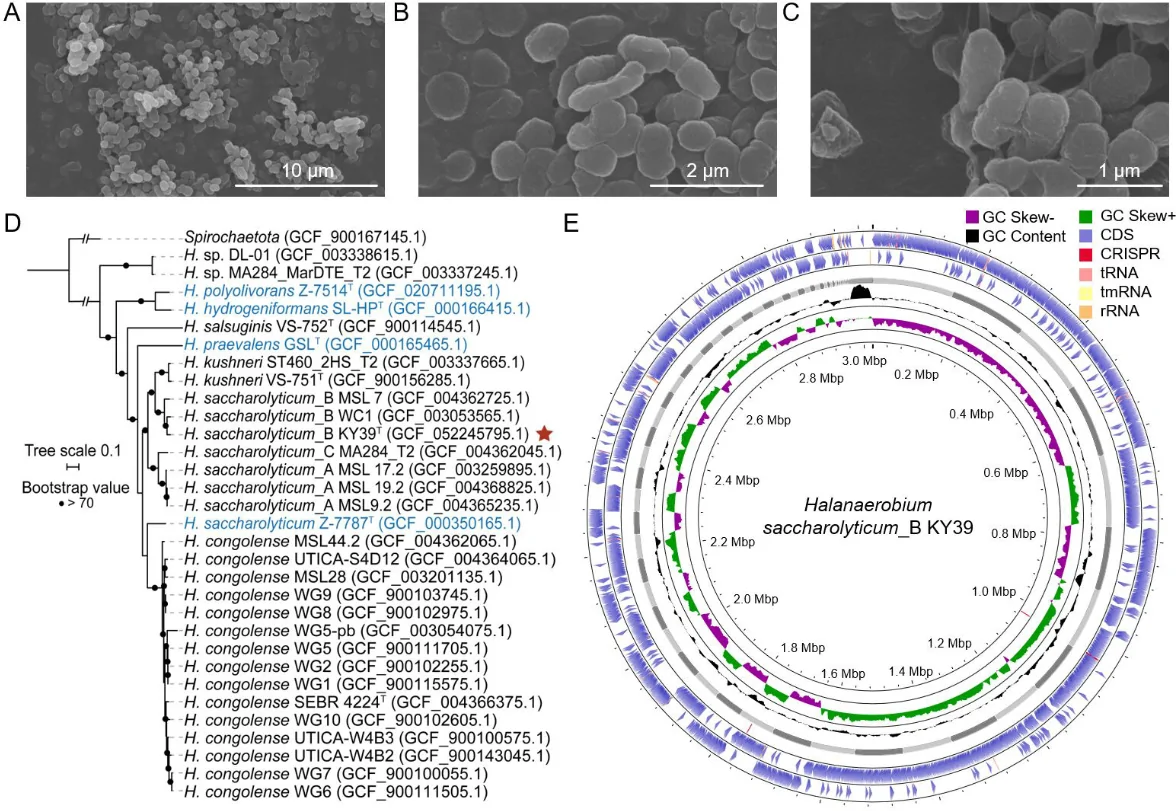
Morphological and genomic characterization of strain KY39. (**A–C**) Scanning electron microscopy (SEM) images of strain KY39. (**D**) Phylogenomic tree based on 31 *Halanaerobium* genomes, including the newly isolated strain KY39 (highlighted with a red star), and 30 genomes for its phylogenetic relatives retrieved from NCBI. A genome from the phylum *Spirochaetota* (GCF_900167145.1) was used as the outgroup. The genomes derived from saline lakes were shown in blue, while the others were originated from oil and gas reservoirs. The type strains were indicated by the superscript “T.” Bootstrap values were calculated from 1,000 replicates, and the filled circles at the nodes indicated the bootstrap values >70%. The scale bar represented 0.1 substitutions per nucleotide position. (**E**) Circular representation of the KY39 genome. From the innermost to the outermost rings: GC skew− (purple), GC skew+ (green), GC content (black), and predicted coding sequences (blue). CRISPR loci, tRNA genes, tmRNA genes, and rRNA genes were shown in red, pink, yellow, and orange, respectively.

A strain designated KY39 was enriched and isolated from the production fluid collected from an oil well in the Zhongyuan Oilfield using the UGW and oUGW media supplemented with 10% salinity. Observation under scanning electron microscopy showed ovoid to short rod-shaped KY39 cells, approximately 0.8–1.2 μm in length and 0.2–0.4 μm in width (Fig. 1A through C). Based on 16S rRNA gene sequence analysis, KY39 exhibited 94.42–99.87% similarity to members of the genus *Halanaerobium*. It exhibited the highest identity (99.87%) to *H. saccharolyticum_*B strains WC1 and MSL7, both isolated from the Utica Shale Formation, USA (Fig. S1; Table S3) (12).

To clarify its taxonomic position, a phylogenomic tree was constructed using available *Halanaerobium* genomes (Fig. 1D). Strain KY39 formed a distinct clade with *H. saccharolyticum*_B strain WC1 with an ANI value of 97.2%, whereas the ANI values between KY39 and other *Halanaerobium* species ranged from 83.2% to 91.3% (Fig. S2; Table S4). The digital DNA-DNA hybridization (dDDH) analysis showed 73.0% similarity between KY39 and WC1, surpassing the 70% threshold for species delineation (54). Collectively, these results support the classification of KY39 as a strain within *H. saccharolyticum*_B clade. The genome of KY39 exhibited a GC content of 36.4% and comprised 2,826 predicted protein-coding sequences, 55 tRNA genes, 1 tmRNA gene, and 9 rRNA genes, which included 6 5S rRNAs, 2 23S rRNAs, and 1 16S rRNA. In addition, two CRISPR-Cas systems were identified (Fig. 1E).

### Physiology and growth characteristics of strain KY39

**Fig 2:**
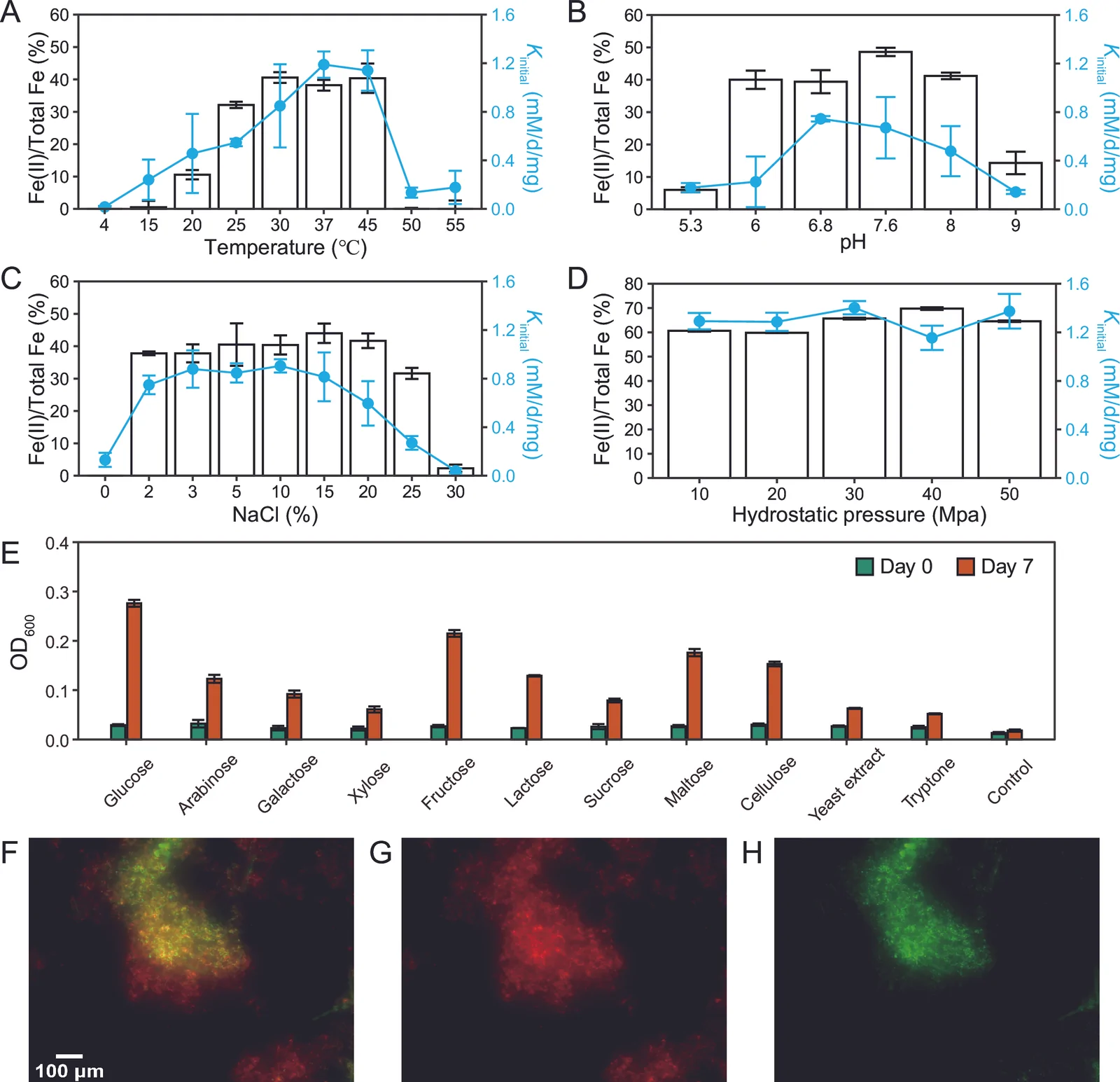
Physiological characteristics and substrate utilization of strain KY39. (**A–D**) Iron reduction by KY39 under different environmental conditions using 10 mM sodium pyruvate as the electron donor and 10 mM ferric citrate as the electron acceptor: (**A**) temperature, (**B**) pH, (**C**) salinity, and (**D**) hydrostatic pressure. In panels **A–D**, the bar charts indicated the ratio of Fe(II) to total iron on day 7, while blue curves represented the initial pseudo-zero order Fe(III) reduction rate constants normalized by protein concentrations. (**E**) Substrate utilization profile of KY39 based on optical density at 600 nm (OD_600_) measured in the presence of various carbohydrates (10 mM each) or complex nutrients (yeast extract and tryptone, 5 g/L) on days 0 and 7. A substrate-free control was developed to exclude the potential contribution from the residual substrates in the medium or introduced from the parental culture. The data in panels **A–E** represented the mean values from three independent biological replicates, and the error bars indicate standard deviation of replicates. (**F–H**) Fluorescence microscope images of strain KY39 stained for live/dead discrimination after cultivation at 37°C with 10 mM sodium pyruvate as the electron donor and 10 mM ferrihydrite as the electron acceptor. Live cells were stained green with Calcein AM, whereas dead cells were stained red with propidium iodide. (**F**) Merged image showing both live (green) and dead (red) cells within aggregates. (**G**) Red fluorescence channel indicating dead cells. (**H**) Green fluorescence channel indicating live cells.

Physiological characterization showed that strain KY39 was a strict anaerobe (Fig. S3). It grew within a temperature range of 20–45°C, with optimal growth at 37°C (Fig. 2A). No iron-reducing activity was detected at temperatures below 20°C or above 45°C. Strain KY39 could tolerate a broad pH range from 5.3 to 9.0, with an optimum at pH 7.6 (Fig. 2B). Salt tolerance assays revealed that the strain grew robustly in media supplemented with 2–30% NaCl (wt/vol), showing optimal growth at 10% NaCl (Fig. 2C). Moreover, KY39 maintained substantial iron-reducing activity under elevated hydrostatic pressure conditions of up to 50 MPa (Fig. 2D).

Strain KY39 could ferment a variety of carbohydrates, including glucose, arabinose, galactose, xylose, fructose, lactose, sucrose, and maltose (Fig. 2E; Table 1). When incubated with 10 mM ferrihydrite as the electron acceptor and 10 mM sodium pyruvate as electron donors, KY39 formed viscous aggregates with the residual iron mineral(s). Fluorescence microscopy revealed both live and dead cells in the aggregates, indicating spatial heterogeneity in cell viability within the aggregates (Fig. 2F through H). When ferric citrate served as the electron acceptor, strain KY39 utilized pyruvate and mannitol as electron donors (Fig. S4). In contrast, small-molecule organic acids (e.g., formate, acetate, and lactate), alcohols (e.g., methanol and ethanol), and hydrogen that commonly act as electron donors for iron reducers did not support its growth.

**TABLE 1:** Comparison of KY39^T^ with other species of the genus *Halanaerobium*^*a*^

Characteristic	*H. saccharolyticum*_B KY39^T^ (this study)	*H. kushneri* VS-751^T^ (55)	*H. salsuginis* VS-752^T^ (56)	*H. congolense* SEBR 4224^T^ (21)	*H. polyolivorans* Z-7514^T^ (57)	*H. saccharolyticum* Z-7787^T^ (25)	*H. praevalens* GSL^T^ (58)	*H. hydrogeniformans* SL-HP^T^ (59)
Cell size (μm)	0.2–0.4 × 0.8–1.2	0.5–0.8 × 0.7–3.3	0.3–0.4 × 2.6–4.0	0.5–1.0 × 2.0–4.0	0.4–0.8 × 1.0–2.0	0.5–0.7 × 1.0–1.5	0.9–1.1 × 2.0–2.6	1.0 × 18.0
Motility	+	+	−	−	−	+	−	−
NaCl concentration (%)	2–30	9–18	6–24	4–24	3.5–22.9	3–30	2–30	2.5–15
Optimum NaCl (%)	10	12	9	10	9.4–12.3	10	13	7
Temperature range (°C)	20–45	20–45	22–51	20–45	14–51	15–47	5–60	20–37
Optimum temperature (°C)	37	35–40	40	42	31–35	37–40	37	33
pH Range	5.3–9.0	6.0–8.0	5.6–8.0	6.3–8.5	6.7–10.1	6.0–8.0	6.0–9.0	7.5–12
Optimum pH	7.6	6.5–7.5	6.1	7.0	8.0–8.5	7.5	7.0–7.4	11.0
Use of substrates								
Arabinose	+	+	+	−	−	+	ND	+
Cellobiose	+	+	−	ND	+	+	−	+
Fructose	+	+	+	+	+	+	+	ND
Galactose	+	+	+	+	+	+	−	+
Glucose	+	+	+	+	+	+	+	+
Glycerol	−	−	−	ND	+	+	−	+
Lactose	+	+	+	−	−	+	−	ND
Maltose	+	+	+	+	ND	+	ND	ND
Pyruvate	+	+	+	ND	+	+	−	ND
Sucrose	+	+	+	+	+	+	−	ND
Xylose	+	−	+	−	+	+	−	+

^*a*^ ND, not determined; +, supported growth; − did not support growth.

FAMEs analysis revealed that the major fatty acids of strain KY39 were C_16:1_ ω9c (39.58%), C_16:1_ ω7c (14.47%), C_16:1_ ω11c (11.76%), and C_14:0_ (10.29%). Unsaturated fatty acids accounted for 65.81% of the total fatty acid content, suggesting that the membrane composition was adapted to hypersaline environments (60). The polar lipid profile consisted of unidentified aminophospholipids (UAPL), unidentified phospholipids (UPL1-3), and unidentified lipids (UL1-7).

### Phylogenetic and genomic traits of the *Halanaerobium* species

All high-quality *Halanaerobium* genomes (completeness >90% and contamination <5%) analyzed in this study were derived from hypersaline environments, including oil and gas reservoirs (*n* = 27) and saline lakes (*n* = 4). Taxonomic assignment and phylogenetic reconstruction placed these genomes into distinct species, including *H. polyolivorans*, *H. hydrogeniformans*, *H. salsuginis*, *H. praevalens*, *H. kushneri*, *H. congolense*, and *H. saccharolyticum* (Fig. 1D). Interestingly, phylogenetic analyses revealed distinct lineage-specific divergence between species derived from oil and gas reservoirs and salt lakes. Except for *H. saccharolyticum* Z-7787, isolated from the biomat of the lagoon in the southern part of the Arabat Strait, East Crimea, *H. congolense* and the reported *H. saccharolyticum*_A-C were recovered from deep subsurface reservoirs (14, 25). For *H. hydrogeniformans*, *H. praevalens*, and *H. polyolivorans*, however, they were derived from saline lakes (Fig. 1D) (57–59). These results suggest that the phylogeny of *Halanaerobium* species may be influenced by their habitats.

Genomic characteristics showed substantial differences between phylogenetic clades of the genus *Halanaerobium*, likely reflecting adaptations to distinct geochemical niches. The genome size for *Halanaerobium* spp. ranged from 2.31 to 3.39 Mb, with GC contents between 30.3% and 36.4%, and gene-coding densities from 87.4% to 90.1% (Table S2). Statistical differences in genome size, coding density, and amino acid composition were observed between genomes from deep reservoirs and saline lakes (Fig. S5; Table S5). Compared to genomes for the *Halanaerobium* species residing in salt lakes, those for the members inhabiting the deep subsurface exhibited significantly higher proportions of proline and tryptophan, but lower levels of isoleucine. Moreover, the amino acid composition was strongly correlated with GC content, showing negative correlation with lysine, isoleucine, asparagine, and cysteine, while positive association with histidine, tyrosine, serine, proline, methionine, phenylalanine, tryptophan, valine, aspartic acid, glycine, arginine, and glutamic acid (Fig. S6; Table S6). Collectively, these results highlight the phylogenetic and genomic differences among *Halanaerobium* lineages associated with distinct habitats.

### Pangenome analyses of *Halanaerobium* species

**Fig 3:**
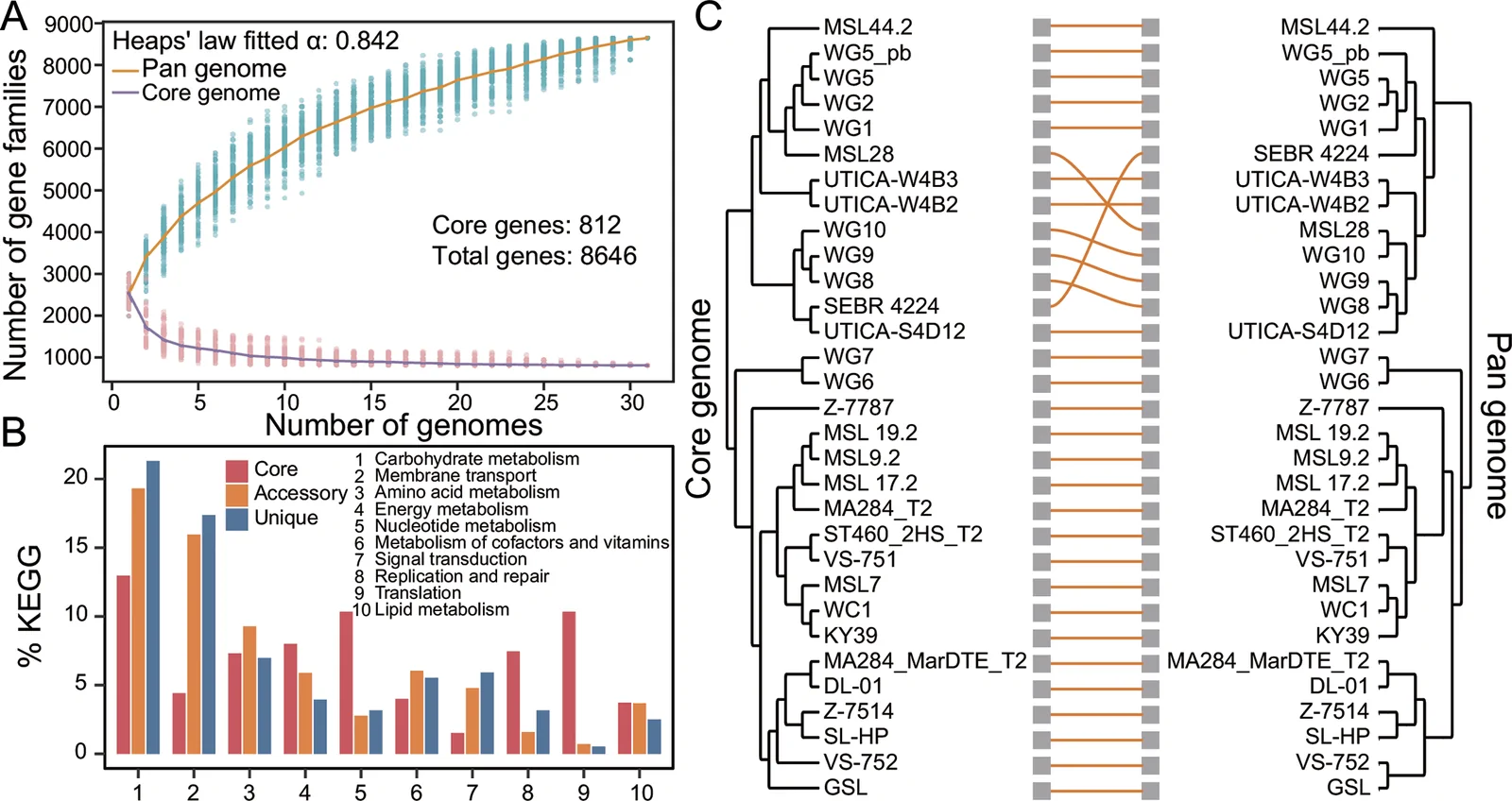
Pangenome analysis of the *Halanaerobium* genomes. (**A**) Pangenome model of the genus *Halanaerobium*, including the Heaps’ law fit parameters (α value), core genome size, and pan-genome size. The order of genome input was provided in Table S7. (**B**) The KEGG category distribution of core, accessory, and unique genes across *Halanaerobium* genomes. Core genes were defined as those shared by all the genomes under study, accessory genes as those present in a subset of genomes, and unique genes as those detected in only one strain. (**C**) Tanglegram analysis comparing the topologies of the phylogenomic trees based on the core genomes (left) and the pangenome (right), respectively. The core-genome tree was constructed based on the single-copy core orthologs, while the pangenome tree was generated from a gene presence/absence matrix. The same genomes between the two trees were connected by lines.

Pangenome analysis of 31 high-quality *Halanaerobium* genomes using BPGA identified a total of 8,646 non-redundant genes (Fig. 3A; Table S7). Annotation of the predicted ORFs based on the KEGG database revealed that most of the genes could be assigned to specific functional categories, with carbohydrate metabolism (17.9%) being the most represented, followed by membrane transport (12.6%), amino acid metabolism (7.9%), and energy metabolism (5.9%) (Fig. 3B; Table S8 through S10). Among these, 812 genes were conserved across all the genomes, comprising the core genome and accounting for approximately 9.4% of the total gene repertoire (Fig. 3A). Meanwhile, the number of strain-specific genes varied markedly among individual genomes, ranging from 0 to 518 (Table S7).

*Halanaerobium* exhibited an open pangenome (Heaps’ law fit parameters *α* = 0.842), as evidenced by the increase in total gene counts with the inclusion of additional genomes. The number of core genes, however, plateaued with the addition of more genomes, indicating a stable and conserved core genome within the genus (Fig. 3A). KEGG annotation showed that 655 out of 812 core genes (80.7%) were assigned to functional categories, with carbohydrate metabolism (13.0%), nucleotide metabolism (10.3%), and translation (10.3%) being the most abundant (Table S8). To further investigate the phylogenetic implications of different gene categories, phylogenetic trees for both the core genome (based on concatenated single-copy core genes) and the pangenome (based on the presence/absence matrix of all genes) were constructed. The tanglegram analysis revealed a high degree of topological congruence between the two trees, with only minor rearrangements observed within individual clades (Fig. 3C). These findings highlight overall inter-strain genomic similarity while supporting clade-specific diversification.

### Metabolic reconstruction of *Halanaerobium* species

**Fig 4:**
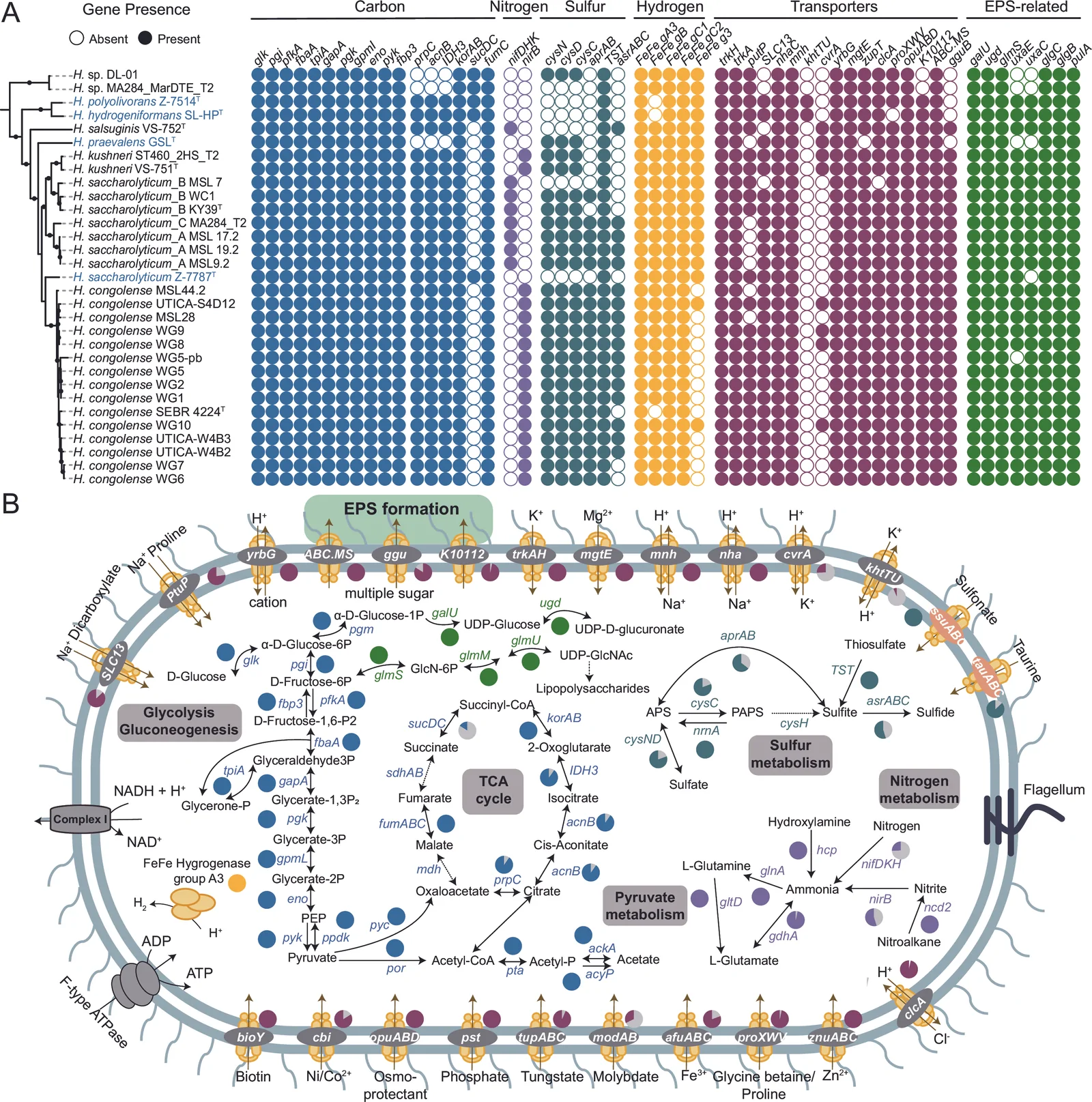
Metabolic features of the genus *Halanaerobium* based on comparative genomic analyses. (**A**) Distribution of genes involved in key metabolic pathways (e.g., carbon, nitrogen, and sulfur metabolism) across 31 high-quality *Halanaerobium* genomes. The phylogenomic tree on the left was consistent with that shown in Fig. 1D. The presence or absence of each gene was represented by filled or open circles, respectively. (**B**) Metabolic reconstruction of *Halanaerobium* spp. For each functional gene, a pie chart adjacent to its label indicated the frequency of occurrence across the 31 *Halanaerobium* genomes. Acetyl-P, acetyl phosphate; APS, adenosine 5′-phosphosulfate; EPS, extracellular polymeric substance; PAPS, 3′-phosphoadenosine 5′-phosphosulfate; PEP, phosphoenolpyruvate; TCA, tricarboxylic acid cycle. The details of the corresponding genes labeled in the diagram are provided in Table S11.

To explore the metabolic potential and ecological versatility of the genus *Halanaerobium*, all 31 high-quality genomes were analyzed to reconstruct key pathways involved in biogeochemical cycling, stress tolerance, and environmental adaptation (Fig. 4A; Table S11). The results revealed a predominantly heterotrophic lifestyle with considerable metabolic versatility across the genus. In addition to phylogenetic clustering, comparative genomic analyses suggested functional differentiation among *Halanaerobium* species associated with their habitats (i.e., oil and gas reservoirs vs saline lakes). PCoA based on the presence/absence of functional genes showed a separation between reservoir- and saline lake-derived genomes (*R*^2^ = 0.094, *P* = 0.015, Fig. S7), suggesting that the functional gene repertoires of *Halanaerobium* spp. may be influenced by their native habitats.

All *Halanaerobium* genomes possessed complete glycolytic and gluconeogenic pathways, whereas the tricarboxylic acid (TCA) cycle was incomplete due to the absence of succinate dehydrogenase (*sdh*) and malate dehydrogenase (*mdh*), indicating limited capacity for complete oxidative metabolism. Only a subset of genomes (e.g., *H. saccharolyticum* Z-7787 and *H. hydrogeniformans* SL-HP) contained *sucCD*, which encodes succinyl-CoA synthetase, indicating restricted interconversion between succinate and succinyl-CoA in certain lineages (Fig. 3B). Differences in substrate utilization were further supported by comparative analyses of carbohydrate-active enzymes and peptidases, which varied across phylogenetic clades (Fig. S8; Table S12 and S13). Notably, the *H. saccharolyticum*_A-C genomes recovered from deep subsurface reservoirs possessed a broader diversity and higher number of carbohydrate-active enzymes and peptidases than other clades, indicating enhanced capacities for carbohydrate and protein degradation in subsurface environments.

Nitrogen metabolic potential also varied among *Halanaerobium* species. Eight genomes harbored the nitrogenase gene cluster *nifDHK*, suggesting their potential for diazotrophic activity, whereas genes involved in dissimilatory nitrate reduction were largely absent across the genus (Fig. 4). In contrast, genomes lacking *nifDHK* frequently contained *nirB*, which mediates dissimilatory nitrite reduction, implying alternative nitrogen acquisition strategies. Notably, *nifDHK* and *nirB* were detected exclusively in a subset of deep subsurface-derived strains and were absent from the saline lake-derived genomes, suggesting that these nitrogen-transforming capabilities may confer a selective advantage under nitrogen-limited conditions in oligotrophic subsurface reservoirs (61–63).

For sulfur metabolism, all *Halanaerobium* genomes possessed genes associated with thiosulfate or sulfite metabolism (e.g., thiosulfate sulfurtransferase, *TST* and anaerobic sulfite reductase, *asrA*), but lacked genes involved in dissimilatory sulfate reduction (e.g., *dsrAB*) and sulfur-oxidizing pathways (e.g., SOX system). Genes involved in anaerobic sulfate and sulfite reduction, including sulfate adenylyltransferase (*cysND*), adenylylsulfate reductase (*aprAB*), and *asrA*, were predominantly present in reservoir-derived genomes, suggesting an increased reliance on sulfur compound transformations for biosynthetic sulfur acquisition in deep subsurface environments.

Genomic evidence further highlighted the widespread presence of genes associated with ion transport and osmoregulation (Fig. 4; Table S11). All *Halanaerobium* strains possessed potassium uptake systems (*trkAH*), sodium/proline symporters (*putP*), and sodium-dependent dicarboxylate transporters (*SLC13*), along with multiple antiporter systems, including Na^+^/H^+^ antiporters (*nha* and *mnh*), K^+^/H^+^ antiporters (*khtTU* and *cvrA*), and a putative Na^+^/Ca^2+^ exchanger (*yrbG*). In addition, genes encoding chloride channels (*clcA*) and osmoprotectant transporters (*proXWV* and *opuABD*) were also broadly distributed, indicating that tolerance to hypersaline conditions by *Halanaerobium* species is associated with both inorganic ion regulation and organic osmolyte accumulation.

Finally, all *Halanaerobium* genomes harbored conserved pathways for activated sugar nucleotide biosynthesis, including glucose-1-phosphate uridylyltransferase (*galU*), UDP-glucose 6-dehydrogenase (*ugd*), glutamine-fructose-6-phosphate transaminase, phosphoglucosamine mutase, and UDP-*N*-acetylglucosamine pyrophosphorylase (*glmSMU* gene cluster). Specifically, *galU* catalyzes the formation of UDP-glucose, a universal precursor for polysaccharide synthesis, while *ugd* converts UDP-glucose to UDP-glucuronate, a key substrate for exopolysaccharide and cell wall polysaccharide formation (64). The *glmSMU* cluster mediates the production of UDP-*N*-acetylglucosamine, another activated sugar required for EPS and peptidoglycan formation (65). In addition, genes involved in glycogen biosynthesis (*glgBC*) and pullulanase (*pulA*) were also conserved across the genus, suggesting potential roles in intracellular carbon storage, carbohydrate recycling, and EPS production (66). Collectively, the presence of these genes suggests that *Halanaerobium* species possess a versatile carbohydrate metabolic network that supports the synthesis of polysaccharide precursors and biofilm formation, potentially facilitating surface attachment and long-term persistence under hypersaline and anoxic conditions.

Taken together, these results indicate that *Halanaerobium* species share a conserved core metabolic framework adapted to hypersaline and anaerobic environments, whereas variation in auxiliary metabolic traits, including carbon utilization, nitrogen, and sulfur metabolism, is broadly associated with their environmental origin. Such patterns are in line with relatively independent adaptive trajectories in distinct extreme habitats, which have been reported for other microbial lineages (67–69). Further studies incorporating expanded genome sampling from saline lakes and experimental validation under controlled environmental gradients will help clarify the ecological significance and potential fitness trade-offs associated with these genomic repertoires.

### The ecological distribution of the genus *Halanaerobium*

**Fig 5:**
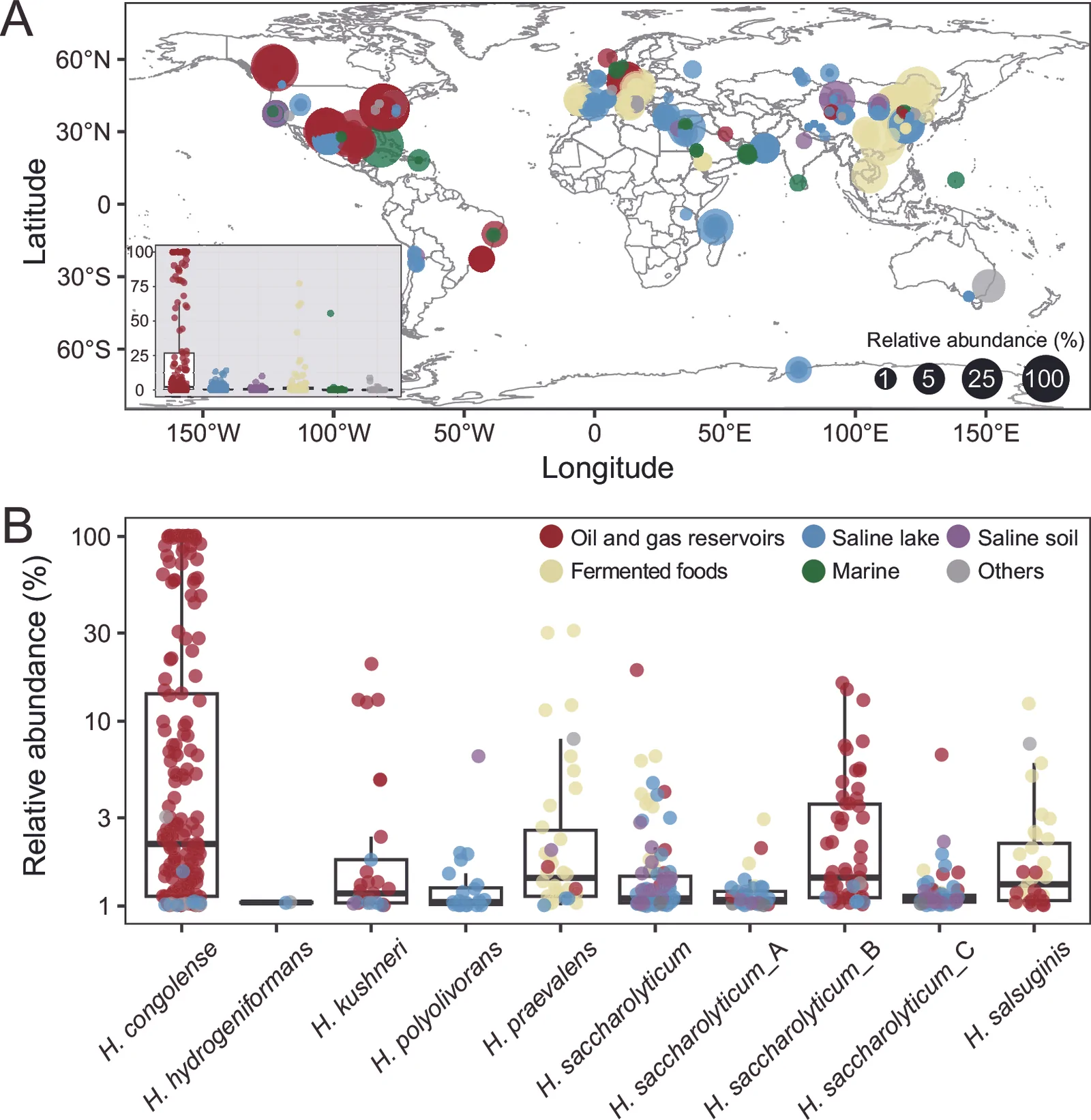
Geographic distribution of the genus *Halanaerobium*. (**A**) Global distribution of *Halanaerobium* species. The presence of the genus *Halanaerobium* was identified by querying the Sandpiper database (53). The point size represented the relative abundance of *Halanaerobium* in each metagenomic data set, while the colors indicated the types of environments from which each metagenome was derived. The subfigure shows the relative abundance of *Halanaerobium* species across different environments. (**B**) Relative abundance of individual *Halanaerobium* species detected in globally distributed metagenomes was determined based on the Sandpiper database (53). Others indicate that environmental information for the corresponding metagenomes was not available.

To further elucidate the ecological distribution of the genus *Halanaerobium*, its environmental occurrence was examined using the integrated Sandpiper metagenomic data sets (53). Members of the genus were detected across geographically broad and ecologically diverse hypersaline environments. In addition to oil and gas reservoirs and saline lakes, they were also found in fermented foods, saline soils, and marine ecosystems (Fig. 5A; Table S14). Notably, *Halanaerobium* species dominated the microbial community in the oil and gas reservoir samples from West Virginia and Ohio, USA, with relative abundances approaching 100% (Fig. 5A). Similarly, high abundances of taxa affiliated to this genus were observed in fermented soybean products, accounting for up to 77.3% of the community. In microbial mats from hypersaline lakes in Spain, members of the genus *Halanaerobium* represented up to 5.7% of the populations (Table S14). By contrast, they generally occurred at low relative abundances (<1%) in most marine samples, except for one sample contaminated by diesel in Key West, Florida, USA (55.56%, Table S14) (70).

While widely distributed, different *Halanaerobium* species exhibited varying preferences for different environmental systems. Specifically, *H. congolense*, *H. saccharolyticum_*B, and *H. kushneri* were predominantly associated with the deep subsurface, whereas *H. hydrogeniformans* and *H. polyolivorans* were mainly enriched in saline lakes (Fig. 5B; Table S15). Although *H. praevalens* and *H. salsuginis* have been cultivated from the surface sediments of the Great Salt Lake and the production fluid from a petroleum reservoir, respectively (56, 58), their close phylogenomic relatives were mainly detected in fermented foods (Fig. 5B). Other different *H. saccharolyticum* clades, however, were detected across a variety of the aforementioned environments and marine habitats (Fig. 5B). Collectively, these results demonstrate that the genus *Halanaerobium* is globally distributed across diverse saline to hypersaline ecosystems, while their environmental preferences or ecological versatility can be species-dependent.

## DISCUSSION

### Adaptation of *Halanaerobium* to saline and hypersaline environments

Culture-dependent and -independent evidence indicates that the genus *Halanaerobium* is broadly distributed across saline to hypersaline environments. The isolated strain KY39 exhibits robust growth under high salinity and elevated pressure, consistent with its subsurface origin and with observations for other *Halanaerobium* spp. (e.g., strains WG8 and DL-01) recovered from deep shale formations (e.g., Utica, Marcellus, and Barnett Shales, USA) (12, 15). Interestingly, although all the available *Halanaerobium* isolates and genomes are derived from saline lakes and oil- and gas-bearing reservoirs, metagenomic surveys suggest a much wider ecological distribution, including saline soils, fermented foods, and marine environments, highlighting a broader habitat range than previously recognized (21, 71, 72).

Adaptation to hypersaline conditions in *Halanaerobium* spp. is primarily achieved through a “salt-in” strategy, supported by coordinated ion transport and homeostasis systems (73, 74). Genomic features indicate active regulation of intracellular K^+^, Na^+^, and Cl^−^ concentrations, together with mechanisms that maintain anion balance and membrane potential (75–77). Divalent cation transporters for Mg^2+^ and Zn^2+^ likely contribute to enzyme stability and metabolic functions under osmotic stress (78). Taken together, these features suggest a tightly regulated osmoregulatory network that enables survival in hypersaline environments.

In addition, accumulation and utilization of compatible solutes (e.g., glycine, betaine, and proline) may stabilize cellular macromolecules and facilitate metabolic interactions within microbial communities (14, 79–83). For example, in Utica and Marcellus shales, betaine produced by the methylotrophic methanogen *Methanohalophilus* may be utilized by *Halanaerobium* spp. to generate trimethylamine, thereby supporting methane production by methanogens (81). The presence of genes related to EPS biosynthesis, consistent with observed cell aggregation, further suggests that biofilm formation may buffer cells against physicochemical fluctuations (19, 20). Collectively, these physiological and structural traits likely support the persistence of *Halanaerobium* spp. under the extreme conditions typical of their habitats.

Beyond stress tolerance, the fermentative metabolism of *Halanaerobium* spp. supports their ecological role in anaerobic carbon, hydrogen, and iron cycling (17, 81). These organisms can utilize a range of carbohydrates, generating metabolites (e.g., acetate, H_2_, and CO_2_) that may sustain syntrophic partners (e.g., methanogens) (84, 85). Notably, *Halanaerobium* may also contribute to Fe(III) reduction, likely mediated by metabolites or electron equivalents generated during fermentation. A similar fermentative iron reduction mechanism has been reported for *Orenia metallireducens* Z6, an isolate from a 2.02 km-deep borehole in the Illinois Basin, USA (86). Fermentative iron reduction by Z6 can modulate the ambient geochemical conditions (e.g., pH) and secondary mineral formation under different environmental conditions (26, 87). These findings suggest that fermentative iron reducers may inhabit deep subsurface environments and catalyze coupled iron and carbon transformation. In addition, partial sulfur compound transformations (thiosulfate to sulfide) may link *Halanaerobium* to sulfur cycling in hypersaline systems, highlighting its potential roles in sustaining trophic interactions within deep biosphere communities (15, 18, 88).

### Habitat-associated genomic differentiation of *Halanaerobium* species

Comparative genomic analyses of currently available high-quality *Halanaerobium* genomes from oil and gas reservoirs and saline lakes suggest habitat-associated functional differentiation. The open pangenome of *Halanaerobium* reflects substantial genomic plasticity, supporting ecological versatility across saline to hypersaline environments (89, 90).

The observed enrichment of genes involved in alanine, aspartate, and glutamate metabolism, as well as lysine degradation, is consistent with adaptation to open and evaporative lake systems characterized by fluctuating salinity and episodic organic matter inputs (91). Under these conditions, amino acid catabolism may facilitate rapid utilization of transient nutrient pulses, in line with the dynamic biogeochemical conditions and high microbial diversity commonly observed in saline lakes (92–96).

In contrast, deep subsurface-derived strains tend to possess larger genomes with expanded transporter and metabolic capacities, suggesting enhanced substrate utilization and metabolic flexibility under chronically energy-limited conditions (97, 98). Specifically, peptidoglycan biosynthesis-related genes may enhance cell wall stability under high-pressure and/or hypersaline conditions, while diverse ABC transporters targeting fermentable substrates (e.g., xylose, rhamnose, and maltose) may support the utilization of organic compounds introduced during hydraulic fracturing activities (99, 100). In addition, genes involved in porphyrin metabolism may enable anaerobic redox reactions critical for energy acquisition in oxygen-deprived reservoirs (101).

Genomic GC content is a fundamental prokaryotic trait closely linked to environmental adaptation and codon usage bias, which in turn can influence amino acid composition (102–105). Habitat-related differences in amino acid usage may therefore partially reflect underlying GC-content variation. In deep-subsurface *Halanaerobium* genomes, the observed higher proportions of GC-rich amino acids (e.g., proline and tryptophan) align with their relatively higher genomic GC content compared to surface hypersaline lake strains. The increased abundance of proline may contribute to protein stabilization and osmotic balance under high salinity and pressure (80). Similarly, the increased tryptophan may help stabilize proteins and limit water intrusion into their hydrophobic cores under high pressure (106). These patterns highlight evolutionary adaptations in deep-subsurface environments, where slightly higher GC content compared to surface lake counterparts may enhance protein stability under extreme conditions.

Collectively, these observations suggest that *Halanaerobium* species inhabiting distinct saline environments exhibit genomic features likely influenced by habitat-specific constraints, which have been widely reported on other groups of organisms (67–69). Future investigations incorporating broader genome sampling and experimental validation under controlled environmental gradients will be essential to more robustly resolve the ecological and functional significance of these adaptive traits.

### Practical implications of *Halanaerobium* spp.

The physiological resilience and metabolic versatility of *Halanaerobium* spp. suggest that they may be “double-edged sword” in practical applications. Their fermentative metabolism, biofilm-forming capacity, and involvement in sulfur and iron transformations indicate potential utility in certain applications, while simultaneously posing some operational risks (e.g., souring, corrosion, and biofouling) (107–109).

The fermentative metabolism underpins both beneficial and detrimental effects. In microbial enhanced oil recovery (MEOR), generation of organic acids (e.g., acetate and propionate) and gases (e.g., CO_2_ and H_2_) by *Halanaerobium* spp. may reduce oil-water interfacial tension, enhance carbonate dissolution, and increase localized reservoir pressure, thereby facilitating hydrocarbon mobilization (73, 110–112). H_2_ production or establishment of syntrophic interactions with methanogens could potentially enhance gas-driven oil displacement (113). Beyond MEOR, such metabolic versatility may support applications in hypersaline wastewater treatment or biohydrogen production (57, 59, 114). Conversely, acid accumulation may lower pH and promote metabolite cross-feeding with sulfate-reducing microorganisms, stimulating sulfide generation that accelerates reservoir souring and increases corrosion risk and desulfurization costs (19, 88, 115).

Biofilm formation similarly exerts dual impacts. In MEOR, biofilms may selectively block high-permeability zones, redirecting flow toward oil-bearing regions (116). However, EPS may trap metals and particulates, promote mineral precipitation, and clog pore spaces or pipelines, particularly in low-permeability reservoirs, increasing maintenance demands (110, 117, 118). A novel observation in this study is the ability of *Halanaerobium* spp. to reduce ferric iron (Fe^3+^) to ferrous iron (Fe^2+^), a process that may promote corrosion through ferric oxide dissolution and enhanced charge transfer on steel surfaces (119). Dense biofilms formed by these bacteria, however, can create microenvironments enriched in Fe^2+^ while limiting oxygen and ion diffusion, highlighting a complex, bidirectional role of ferric iron reduction in industrial systems (119, 120).

Mitigation strategies, particularly the targeted application of biocide, remain important for controlling *Halanaerobium*-associated risks. Previous work indicates that quaternary ammonium compounds can be effective under thiosulfate-reducing conditions, at lower dosages than conventional biocides (19). Sustainable alternatives (e.g., plant-derived antimicrobials) and approaches exploiting microbial competition may also represent promising options.

Overall, *Halanaerobium* spp. exhibit genomic and physiological traits that may be harnessed in MEOR and other industrial applications, yet their involvement in corrosion, souring, and biofouling necessitates careful management. The beneficial and detrimental effects underscore the importance of integrated strategies that fully consider environmental context and microbial interactions in industrial systems, balancing potential opportunities with operational risks.

### Conclusions

This study provides a comprehensive insight into the biogeography, ecological system-dependent adaptive evolution, and physiology of *Halanaerobium* spp. in saline and hypersaline environments. Comparison of the available *Halanaerobium* isolates demonstrates not only their similarities in adaptation to hypersaline environments, but also divergence in physiological traits for the strain derived from different environments. Genomic analyses of 31 high-quality *Halanaerobium* genomes revealed an open pangenome for this genus, which may facilitate gene acquisition and promote both phylogenetic and metabolic diversification for the strains driven by distinct ecological niches. As the key diazotrophs and fermenters, *Halanaerobium* spp. may play vital roles in nutrient cycling and microbial community stability. The shared and distinct genetic and physiological features of the genus *Halanaerobium* support their wide distribution in a variety of natural and human-interfered saline to hypersaline ecosystems.

Beyond their well-documented roles in oil and gas industries, the metabolic versatility of the genus *Halanaerobium* implies promising industrial potential. For example, their capacity for fermentation has been recognized for the potential of biofuel production and sustainable utilization of organic carbon-rich industrial wastes (121). Promoting growth of the non-sulfidogenic populations (e.g., *Halanaerobium* spp. WG6 and WG7) may suppress sulfidogenic *Halanaerobium* and other taxa, suggesting that resource competition within these communities could be leveraged as a biocontrol strategy in engineered systems. Collectively, these characteristics suggest that *Halanaerobium* spp. are versatile organisms with ecological significance, while their underexplored industrial values are worth further investigation. While our biogeographic survey demonstrates that genus *Halanaerobium* is distributed in a variety of saline to hypersaline environments (Fig. 5). A deeper understanding of their metabolic networks will be essential to harness their ecological roles and beneficial potentials, mitigating their negative impacts, and ultimately supporting sustainable industrial applications and environmental stewardship.

## Supplementary Material

Reviewer comments

## Data Availability

The genomic sequence of strain KY39 has been deposited in the NCBI database under project number PRJNA1308368 with the accession number GCF_052245795.1 and at the National Omics Data Encyclopedia (NODE) under Analysis ID OEZ00021639. Accession numbers of the other 30 high-quality *Halanaerobium* genomes were provided in Table S2.

## SUPPLEMENTAL MATERIAL

Fig. S1 to S8 spectrum.01381-26-s0001.docx Fig. S1: Phylogenetic analysis of strain KY39. Fig. S2: Average nucleotide identity (ANI) analysis among the 31 *Halanaerobium* genomes. Fig. S3: Growth curve of strain KY39. Fig. S4: Electron donor utilization by strain KY39. Fig. S5: Comparison of the genomic characteristics of *Halanaerobium* species derived from different environments. Fig. S6: Correlation between amino acid composition and genomic G+C content. Fig. S7: Differentiated metabolic potential between *Halanaerobium* species. Fig. S8: Distribution of carbohydrate-active enzymes and peptidases across 31 high-quality *Halanaerobium* genomes.

Tables S1 to S15 spectrum.01381-26-s0002.xlsx Table S1: Environmental parameters of production water from the Zhongyuan Oilfield, Henan, China. Table S2: List of all 31 *Halanaerobium* genomes analyzed in this study. Table S3: Sequence identities of 16S rRNA genes between KY39 and other *Halanaerobium* genomes. Table S4: ANI values among all *Halanaerobium* species. Table S5: Amino acid composition across *Halanaerobium* species. Table S6: Correlation between the content of amino acids and genome G+C content. Table S7: Pangenome profiles of 31 high-quality *Halanaerobium* genomes. Table S8: KEGG categories of the pangenome of the genus *Halanaerobium*. Table S9: COG categories of the pangenome of the genus *Halanaerobium*. Table S10: Functional details of the core genome of the genus *Halanaerobium*. Table S11: Functional profiles of 31 high-quality *Halanaerobium* genomes. Table S12: CAZymes detected in the *Halanaerobium* genomes. Table S13: Peptidases detected in the *Halanaerobium* genomes. Table S14: Global distribution and relative abundance of the genus *Halanaerobium* in metagenomes based on the Sandpiper database. Table S15: Global distribution and relative abundance of the *Halanaerobium* species in metagenomes based on the Sandpiper database.

OPEN PEER REVIEW reviewer-comments.pdf An accounting of the reviewer comments and feedback.

ASM does not own the copyrights to Supplemental Material that may be linked to, or accessed through, an article. The authors have granted ASM a non-exclusive, world-wide license to publish the Supplemental Material files. Please contact the corresponding author directly for reuse.
