# Supplementary material for: Ecological plasticity of Halanaerobium microorganisms across terrestrial saline to hypersaline subsurface environments
Source: Microbiol Spectr. 2026 Jun 15;14(7):e01381-26. doi: 10.1128/spectrum.01381-26 (PMC13340142; doi:10.1128/spectrum.01381-26)
Supplement: Fig. S1 to S8 — Fig. S1: Phylogenetic analysis of strain KY39. Fig. S2: Average nucleotide identity (ANI) analysis among the 31 Halanaerobium genomes. Fig. S3: Growth curve of strain KY39. Fig. S4: Electron donor utilization by strain KY39. Fig. S5: Comparison of the genomic characteristics of Halanaerobium species derived from different environments. Fig. S6: Correlation between amino acid composition and genomic G+C content. Fig. S7: Differentiated metabolic potential between Halanaerobium species. Fig. S8: Distribution of carbohydrate-active enzymes and peptidases across 31 high-quality Halanaerobium genomes. [file spectrum.01381-26-s0001.docx]

Supplementary Information for

**Ecological plasticity of *Halanaerobium* microorganisms across terrestrial saline to hypersaline subsurface environments**

Yu He^1,2^, Xinhang Wang^1^, Shuyi Li^1^, Chenxi Zhang^1^, Mingxia Xu^1^, Yushan Zhou^1^, Robert A. Sanford^3^, Renxing Liang^4^, Yanqun Zhu^5^, Dongbo Yang^5^, Lihua Dan^5^, Xingdong Mao^5^, Lei Zhang^5^, Weimin Sun^6^, Yongguang Jiang^1^, Yidan Hu^1^, Zhou Jiang^1^, Yongzhe Li^1, 7^, Wentao Song^1^, Na Hu^1^, Lu Zhao^1^, Yiran Dong^1, 4, 8, 9, 10^*, Liang Shi^1, 4, 8^

^1^ School of Environmental Studies, China University of Geosciences (Wuhan), Wuhan, China

^2^ School of Environment and Resources, Taiyuan University of Science and Technology, Taiyuan, China

^3^ Department of Earth Science & Environmental Change, University of Illinois Urbana-Champaign, IL, U. S.

^4^ State Key Laboratory of Geomicrobiology and Environmental Changes, China University of Geosciences (Wuhan), Wuhan, China

^5^ Sinopec Zhongyuan OilField, Henan, China

^6^ Guangdong Institute of Eco-environmental and Soil Science, Guangdong, China

^7^ Central and South China Municipal Engineering Design and Research Institute Co, Ltd., Wuhan, China.

^8^ State Environmental Protection Key Laboratory of Source Apportionment and Control of Aquatic Pollution, Ministry of Ecology and Environment, Wuhan, China

^9^ Hubei Key Laboratory of Yangtze Catchment Environmental Aquatic Science, Wuhan, China

^10^ Key Laboratory of Groundwater Quality and Health (China University of Geosciences), Ministry of Education, Wuhan, China

* Corresponding author. Mailing address: 388 Lumo Road, Wuhan, China, 430074. Phone: +11(27)67883152. Email: [dongyr@cug.edu.cn](mailto:dongyr@cug.edu.cn)

**This supplementary information includes 16 tables and 7 figures.**

**SUPPLEMENTARY TABLES**

**Please refer to the Excel files for supplementary tables.**

**Table S1.** Environmental parameters of production water from the Zhongyuan Oilfield, Henan, China.

**Table S2.** List of all 31 *Halanaerobium* genomes analyzed in this study.

**Table S3.** Sequence identities of 16S rRNA genes between KY39 and other *Halanaerobium* genomes.

**Table S4.** ANI values among all *Halanaerobium* species.

**Table S5.** Amino acid composition across *Halanaerobium* species.

**Table S6.** Correlation between the content of amino acids and genome G+C content.

**Table S7.** Pangenome profiles of 31 high-quality *Halanaerobium* genomes.

**Table S8.** KEGG categories of the pangenome of the genus *Halanaerobium*.

**Table S9.** COG categories of the pangenome of the genus *Halanaerobium*.

**Table S10.** Functional details of the core genome of the genus *Halanaerobium*.

**Table S11.** Functional profiles of 31 high-quality *Halanaerobium* genomes.

**Table S12.** CAZymes detected in the *Halanaerobium* genomes.

**Table S13.** Peptidases detected in the *Halanaerobium* genomes.

**Table S14.** Global distribution and relative abundance of the genus *Halanaerobium* in metagenomes based on the Sandpiper database.

**Table S15.** Global distribution and relative abundance of the *Halanaerobium* species in metagenomes based on the Sandpiper database.

**SUPPLEMENTARY FIGURES**

**Fig. S1** Phylogenetic analysis of strain KY39. The maximum likelihood tree was constructed based on the full-length 16S rRNA gene sequences of strain KY39 (highlighted in red) and reference *Halanaerobium* genomes. The GenBank accession numbers for the *Halanaerobium* genomes were provided in parentheses.

**Fig. S2** Average nucleotide identity (ANI) analysis among the 31 *Halanaerobium* genomes.

**Fig. S3** Growth curve of strain KY39. The strain was cultured with 10 mM glucose as the fermentation substrate. Error bars indicate the standard deviation of three biological replicates.

**Fig. S4** Electron donor utilization by strain KY39. Fe(II) production was measured in the cultures amended with 10 mM ferric citrate as the electron acceptor and one of the compounds under investigation as the electron donor. Ten mM soluble organic compounds or 5 mL/tube H_2_ were amended. Ferrous iron concentrations were determined on days 0 and 7. Error bars represented the standard deviation for replicate samples.

**Fig. S5** Comparison of the genomic characteristics of *Halanaerobium* species derived from different environments. The significant differences between genomes from oil and gas reservoirs and salt lakes were evaluated using Wilcoxon signed-rank test (* *p* < 0.05).

**Fig. S6** Correlation between amino acid composition and genomic G+C content. Linear regression analysis showing the relationships between the frequencies of each of the 20 amino acids and genomic G+C content across 31 *Halanaerobium* genomes. Statistical significance was assessed by a two-tailed *t*-test on the regression slope, with *p* values < 0.05 considered significant. Detailed statistical results were provided in Table S6.

**Fig. S7** Differentiated metabolic potential between *Halanaerobium* species. (A) PCoA of 31 *Halanaerobium* genomes based on the presence/absence matrix of functional genes. Each point represented a *Halanaerobium* genome recovered from oil and gas reservoirs (red) or saline lakes (blue). The strain KY39 isolated in this study was highlighted with a black frame. The significant difference between the two groups was evaluand by PERMANOVA. (B) Enrichment analysis of differential gene comparing genomes from oil and gas reservoirs versus saline lakes. The x-axis represents normalized enrichment scores, and the y-axis shows the negative common logarithm of the *p*-values associated with the enrichment of a gene set (-log10(*p*)). Selected functional categories enriched in reservoir-derived genomes (red) and saline lake-derived genomes (blue) were labeled.

**Fig. S8** Distribution of carbohydrate-active enzymes and peptidases across 31 high-quality *Halanaerobium* genomes. (A) Principal coordinates analysis (PCoA) based on the distribution of carbohydrate-active enzymes. (B) PCoA based on the distribution of peptidases. In A-B, the significant difference between the genomes from oil and gas reservoirs and saline lakes was evaluand by PERMANOVA. (C) Number of carbohydrate-active enzymes in each category identified in individual *Halanaerobium* genome. The genomes derived from oil and gas reservoirs and saline lakes were labeled in red and blue, respectively. Detailed data was provided in Tables S12 and S13.

**SUPPLEMENTARY FIGURES**


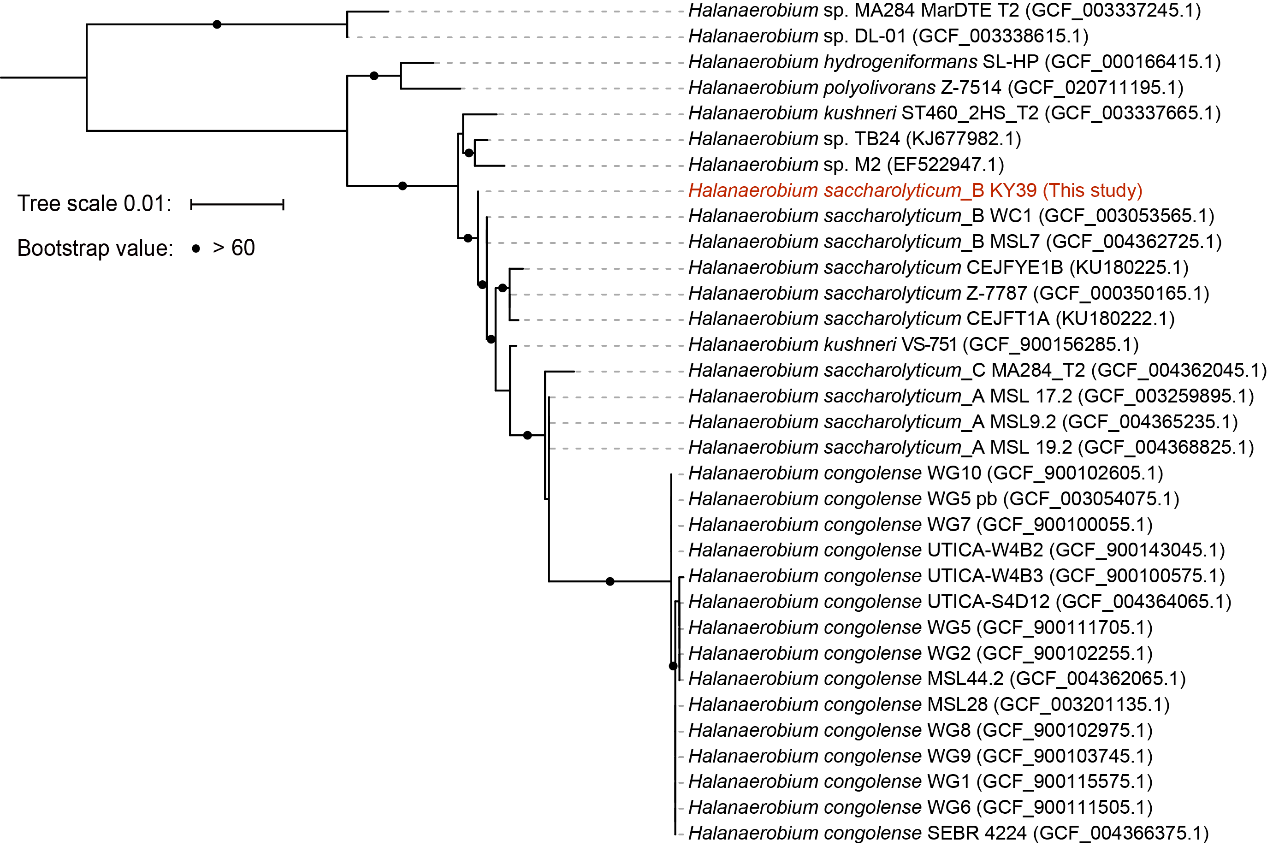


**Fig. S1 Phylogenetic analysis of strain KY39.** The maximum likelihood tree was constructed based on the full-length 16S rRNA gene sequences of strain KY39 (highlighted in red) and reference *Halanaerobium* genomes. The GenBank accession numbers for the *Halanaerobium* genomes were provided in parentheses.


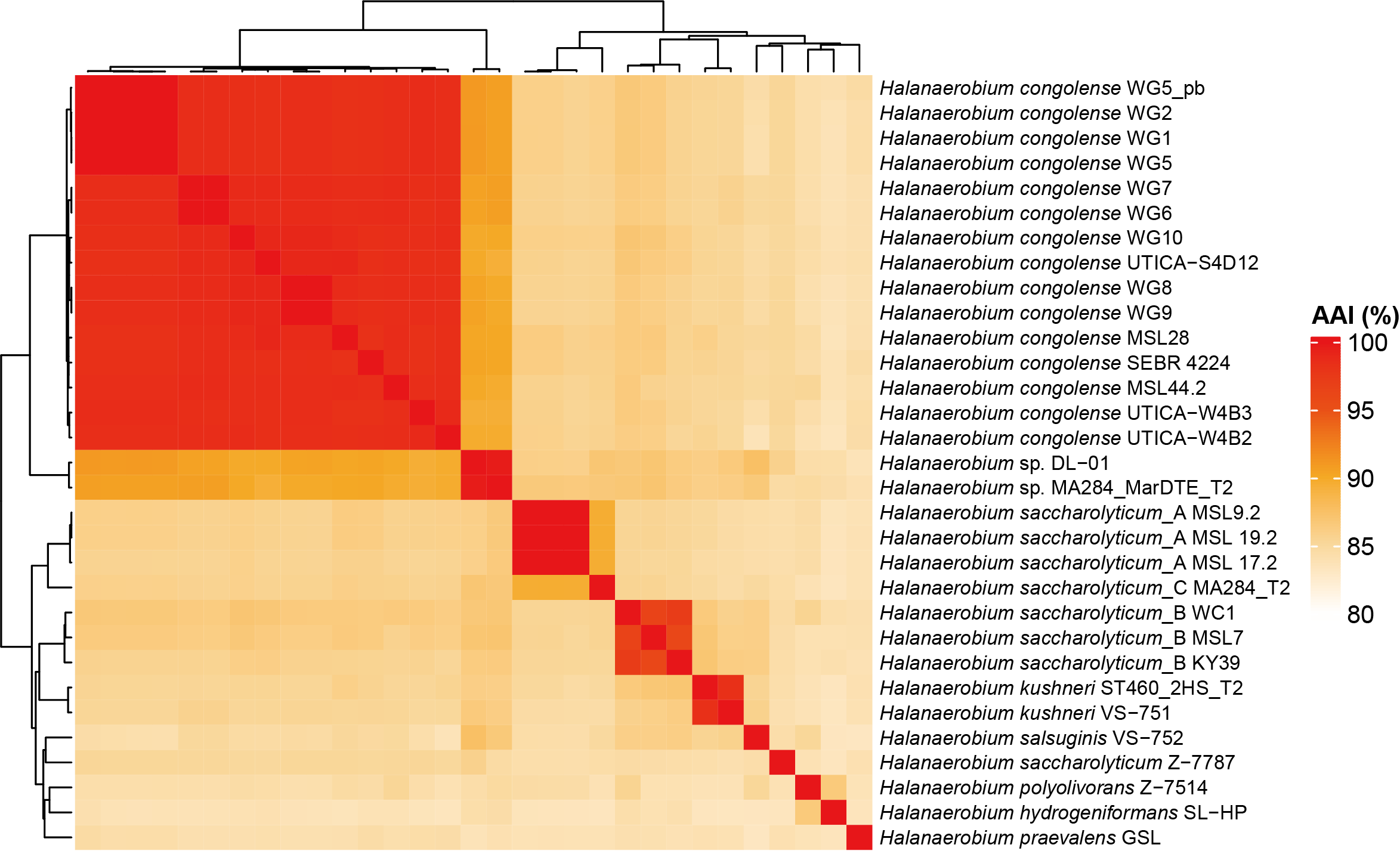


**Fig. S2 Average nucleotide identity (ANI) analysis among the 31 *Halanaerobium* genomes.**


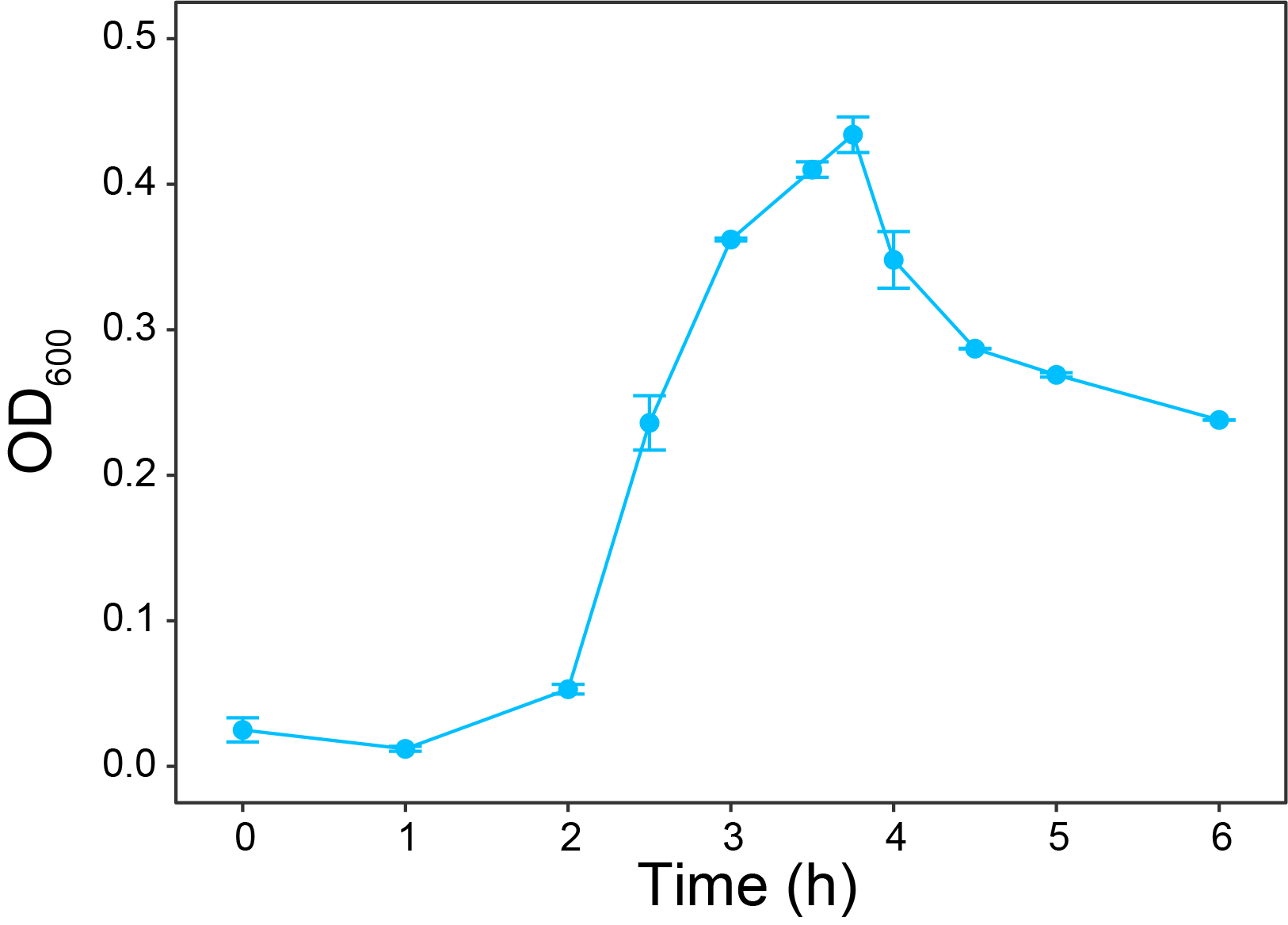


**Fig. S3 Growth curve of strain KY39.** The strain was cultured with 10 mM glucose as the fermentation substrate. Error bars indicate the standard deviation of three biological replicates.


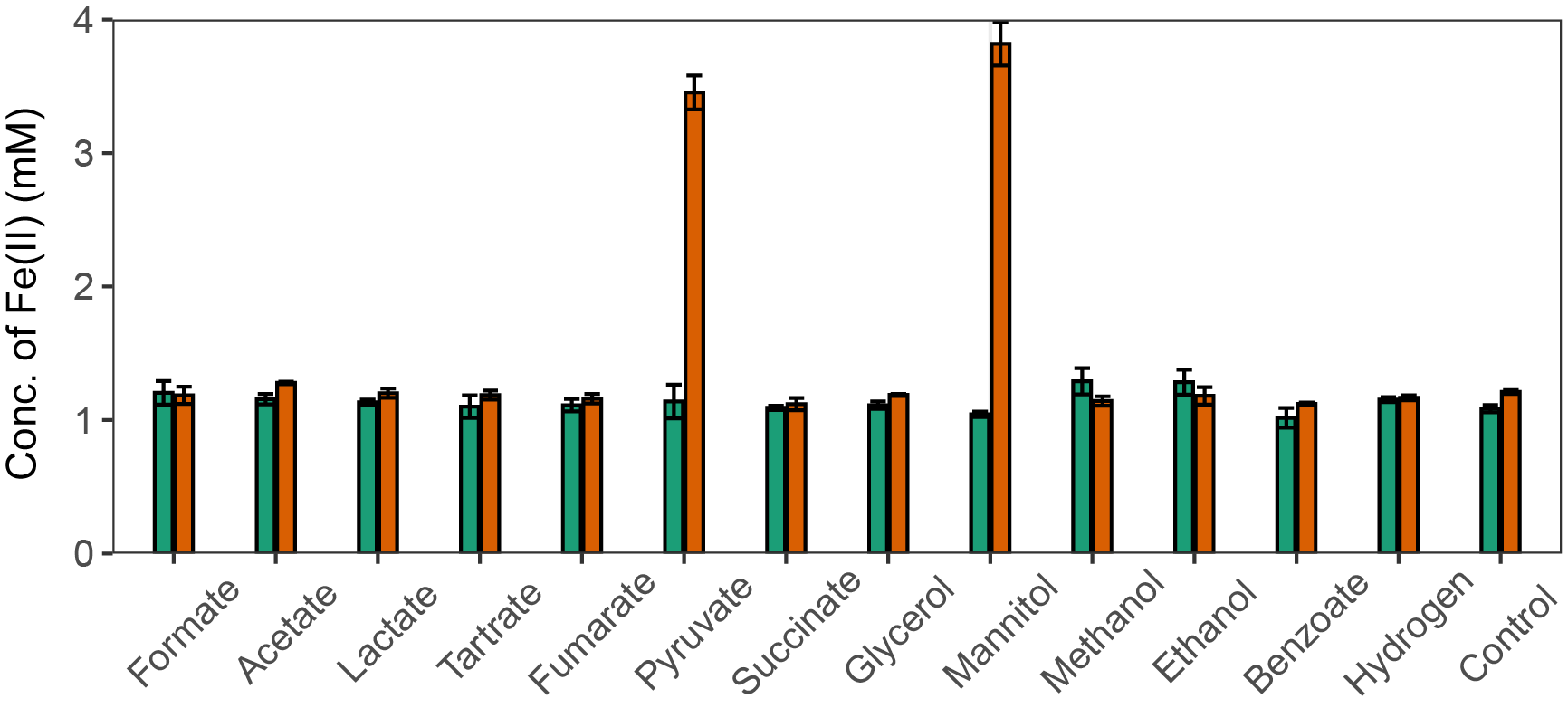


**Fig. S4 Electron donor utilization by strain KY39.** Fe(II) production was measured in the cultures amended with 10 mM ferric citrate as the electron acceptor and one of the compounds under investigation as the electron donor. Ten mM soluble organic compounds or 5 mL/tube H_2_ were amended. Ferrous iron concentrations were determined on days 0 and 7. Error bars represented the standard deviation for replicate samples.


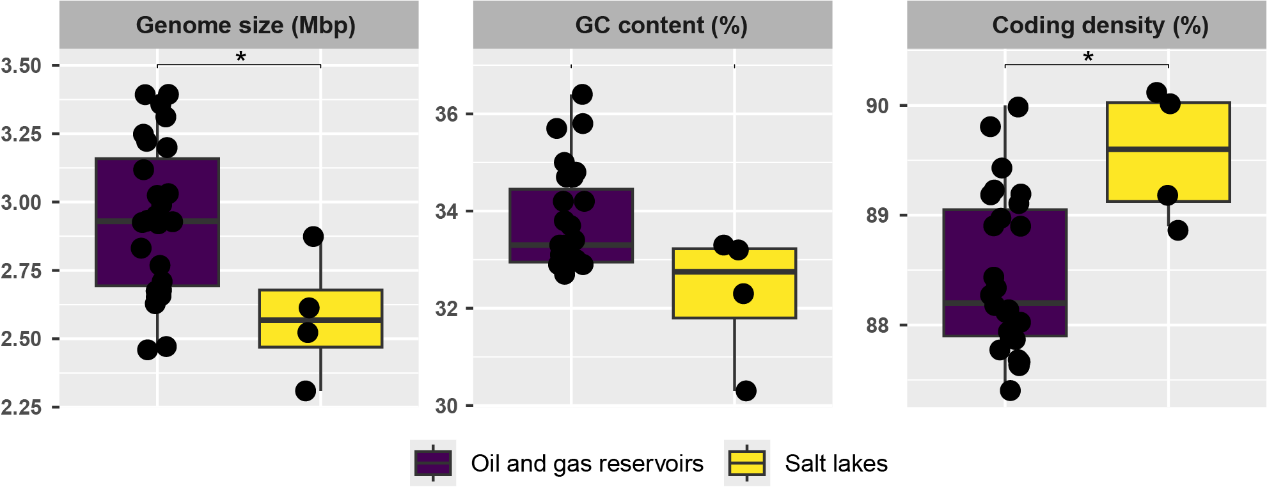


**Fig. S5 Comparison of the genomic characteristics of *Halanaerobium* species derived from different environments.** The significant differences between genomes from oil and gas reservoirs and salt lakes were evaluated using Wilcoxon signed-rank test (* *p* < 0.05).


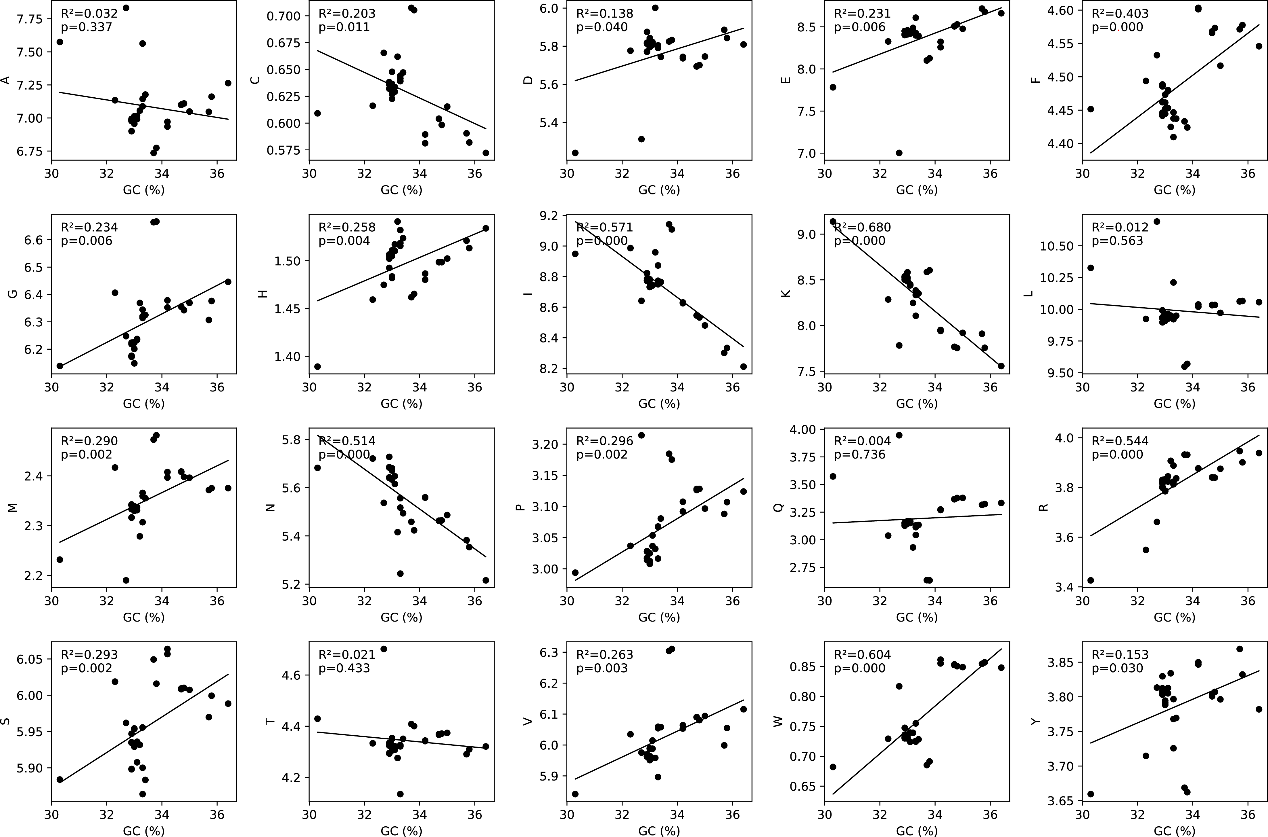


**Fig. S6 Correlation between amino acid composition and genomic G+C content.** Linear regression analysis showing the relationships between the frequencies of each of the 20 amino acids and genomic G+C content across 31 *Halanaerobium* genomes. Statistical significance was assessed by a two-tailed *t*-test on the regression slope, with *p* values < 0.05 considered significant. Detailed statistical results were provided in Table S6.


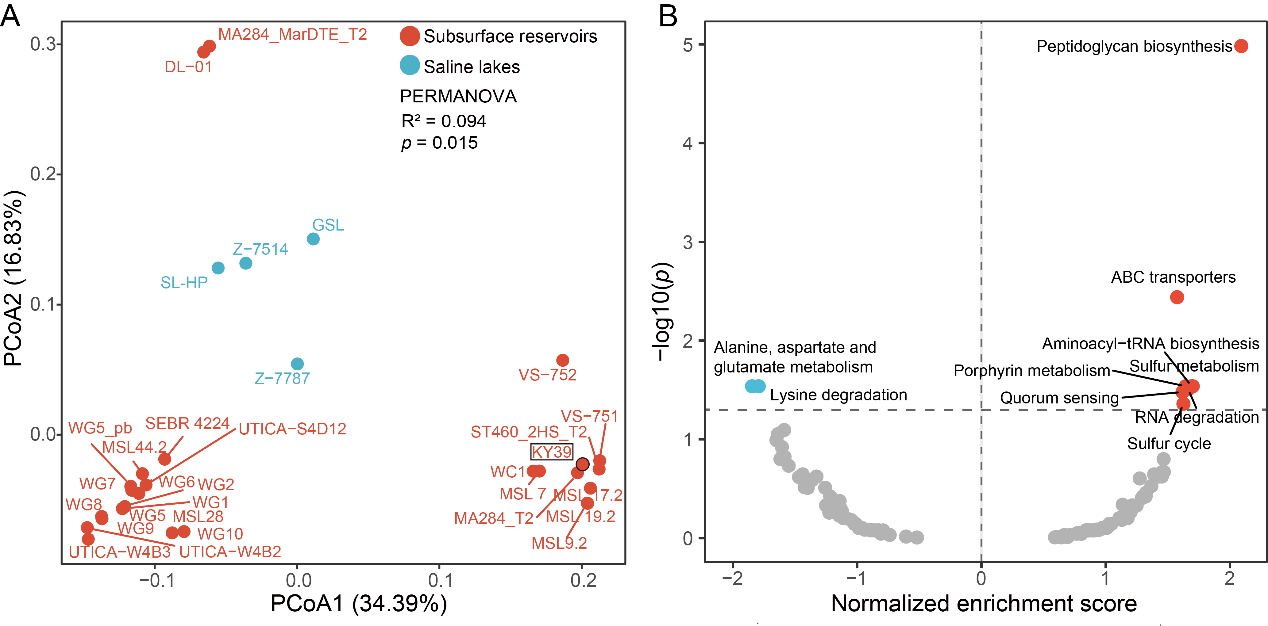


**Fig. S7 Differentiated metabolic potential between** ***Halanaerobium* species.** **(A)** PCoA of 31 *Halanaerobium* genomes based on the presence/absence matrix of functional genes. Each point represented a *Halanaerobium* genome recovered from oil and gas reservoirs (red) or saline lakes (blue). The strain KY39 isolated in this study was highlighted with a black frame. The significant difference between the two groups was evaluand by PERMANOVA. **(B)** Enrichment analysis of differential gene comparing genomes from oil and gas reservoirs versus saline lakes. The x-axis represents normalized enrichment scores, and the y-axis shows the negative common logarithm of the *p*-values associated with the enrichment of a gene set (-log10(*p*)). Selected functional categories enriched in reservoir-derived genomes (red) and saline lake-derived genomes (blue) were labeled.


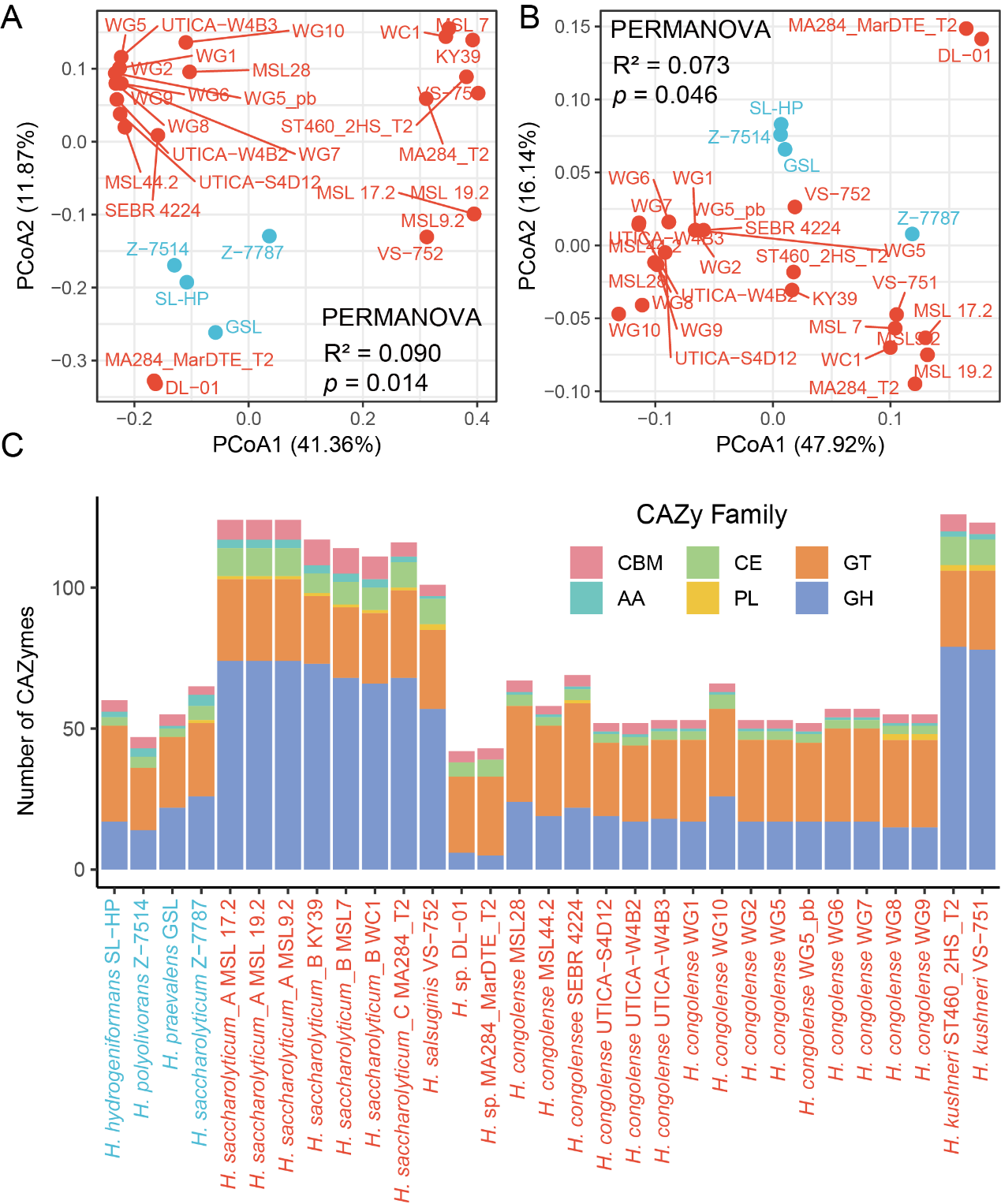


**Fig. S8 Distribution of carbohydrate-active enzymes and peptidases across 31 high-quality *Halanaerobium* genomes. (A)** Principal coordinates analysis (PCoA) based on the distribution of carbohydrate-active enzymes. **(B)** PCoA based on the distribution of peptidases. In A-B, the significant difference between the genomes from oil and gas reservoirs and saline lakes was evaluand by PERMANOVA. **(C)** Number of carbohydrate-active enzymes in each category identified in individual *Halanaerobium* genome. The genomes derived from oil and gas reservoirs and saline lakes were labeled in red and blue, respectively. Detailed data was provided in Tables S12 and S13.
