# Supplementary material for: Ecological plasticity of Halanaerobium microorganisms across terrestrial saline to hypersaline subsurface environments
Source: Microbiol Spectr. 2026 Jun 15;14(7):e01381-26. doi: 10.1128/spectrum.01381-26 (PMC13340142; doi:10.1128/spectrum.01381-26)
Supplement: Reviewer comments [file reviewer-comments.pdf]

AMERICAN SOCIETY FOR MICROBIOLOGY | Microbiology Spectrum

Ecological plasticity of *Halanaerobium* microorganisms across terrestrial saline to hypersaline subsurface environments

Yu He, Xinhang Wang, Shuyi Li, Chenxi Zhang, Mingxia Xu, Yushan Zhou, Robert Sanford, Renxing Liang, Yanqun Zhu, Dongbo Yang, Lihua Dan, Xingdong Mao, Lei Zhang, Weimin Sun, Yongguang Jiang, Yidan Hu, Zhou Jiang, Yongzhe Li, Wentao Song, Na Hu, Lu Zhao, Yiran Dong, and Liang Shi

Corresponding Author(s): Yiran Dong, China University of Geosciences

Review Timeline:

Submission Date:	April 22, 2026
Editorial Decision:	April 30, 2026
Revision Received:	April 30, 2026
Accepted:	May 4, 2026

Editor: Blaire Steven

Reviewer(s): The reviewers have opted to remain anonymous.

Transaction Report:

(Note: With the exception of the correction of typographical or spelling errors that could be a source of ambiguity, letters and reports are not edited. The original formatting of letters and referee reports may not be reflected in this compilation.)

DOI: https://doi.org/10.1128/spectrum.01381-26

Re: Spectrum01381-26 (Ecological plasticity of *Halanaerobium* microorganisms across terrestrial saline to hypersaline subsurface environments)

Dear Dr. Yiran Dong:

Thank you for the privilege of reviewing your work.

After having read the manuscript and the response to reviewer comments I am recommending provisional acceptance of the submission.

I am pleased to inform you that your manuscript has been editorially accepted for publication. However, there are a few additional questions in the submission form that need to be answered before your manuscript is officially accepted. Once these are completed, please return your submission so that I can move your paper forward to acceptance.

Please return the manuscript within 60 days; if you cannot complete the submission questions within this time period, please contact me.

Thank you for submitting your paper to Spectrum.

Sincerely,
Blaire Steven
Editor
Microbiology Spectrum

Re: Spectrum01381-26R1 (Ecological plasticity of *Halanaerobium* microorganisms across terrestrial saline to hypersaline subsurface environments)

Dear Dr. Yiran Dong:

Your manuscript has been accepted, and I am forwarding it to the ASM production staff for publication. Your paper will first be checked to make sure all elements meet the technical requirements. ASM staff will contact you if anything needs to be revised before copyediting and production can begin. Otherwise, you will be notified when your proofs are ready to be viewed.

Data Availability: ASM policy requires that data be available to the public upon online posting of the article, so please verify all links to sequence records, if present, and make sure that each number retrieves the full record of the data. If a new accession number is not linked or a link is broken, provide production staff with the correct URL for the record. If the accession numbers for new data are not publicly accessible before the expected online posting of the article, publication may be delayed; please contact ASM production staff immediately with the expected release date.

Publication Fees: For information on publication fees and which article types have charges, please visit our website. We have partnered with Copyright Clearance Center (CCC) to collect author charges. If fees apply to your paper, you will receive a message from no-reply@copyright.com with further instructions. For questions related to paying charges through RightsLink, please contact CCC at ASM_Support@copyright.com or toll free at +1-877-622-5543. CCC makes every attempt to respond to all emails within 24 hours.

ASM Membership: Corresponding authors may join or renew ASM membership to obtain discounts on publication fees. Need to upgrade your membership level? Please contact Customer Service at Service@asmusa.org.

Embargo Policy: A press release may be issued as soon as the manuscript is posted on the Spectrum Latest Articles webpage. The corresponding author will receive an email with the subject line "ASM Journals Author Services Notification" when the article is available online.

The ASM Journals program strives for constant improvement in our submission and publication process. Please tell us how we can improve your experience by taking this quick Author Survey.

Thank you for submitting your paper to Spectrum.

Sincerely,
Blaire Steven
Editor
Microbiology Spectrum
